# Estimating the Below-Ground Leak Rate of a Natural Gas Pipeline Using Above-Ground Downwind Measurements: The ESCAPE^−1^ Model

**DOI:** 10.3390/s23208417

**Published:** 2023-10-12

**Authors:** Fancy Cheptonui, Stuart N. Riddick, Anna L. Hodshire, Mercy Mbua, Kathleen M. Smits, Daniel J. Zimmerle

**Affiliations:** 1 Department of Systems Engineering, Colorado State University, Fort Collins, CO 80523, USA; 2 Energy Institute, Colorado State University, Fort Collins, CO 80524, USA; 3 Department of Civil and Environmental Engineering, Southern Methodist University, Dallas, TX 75205, USA

**Keywords:** methane, leak detection, leak quantification, pipeline safety, greenhouse gases

## Abstract

Natural gas (NG) leaks from below-ground pipelines pose safety, economic, and environmental hazards. Despite walking surveys using handheld methane (CH_4_) detectors to locate leaks, accurately triaging the severity of a leak remains challenging. It is currently unclear whether CH_4_ detectors used in walking surveys could be used to identify large leaks that require an immediate response. To explore this, we used above-ground downwind CH_4_ concentration measurements made during controlled emission experiments over a range of environmental conditions. These data were then used as the input to a novel modeling framework, the ESCAPE^−1^ model, to estimate the below-ground leak rates. Using 10-minute averaged CH_4_ mixing/meteorological data and filtering out wind speed < 2 m s^−1^/unstable atmospheric data, the ESCAPE^−1^ model estimates small leaks (0.2 kg CH_4_ h^−1^) and medium leaks (0.8 kg CH_4_ h^−1^) with a bias of −85%/+100% and −50%/+64%, respectively. Longer averaging (≥3 h) results in a 55% overestimation for small leaks and a 6% underestimation for medium leaks. These results suggest that as the wind speed increases or the atmosphere becomes more stable, the accuracy and precision of the leak rate calculated by the ESCAPE^−1^ model decrease. With an uncertainty of ±55%, our results show that CH_4_ mixing ratios measured using industry-standard detectors could be used to prioritize leak repairs.

## 1. Introduction

In recent years, natural gas (NG) has been used as an environmentally cleaner energy source compared to coal or oil as its combustion results in the lower emission of greenhouse gas pollutants such as CO_2_ [1]. The US production of NG in 2022 increased by 4% or 4.9 Bcf/d (averaging 119 Bcf/d) as compared to the production in 2021 [2]. Typically, pipelines are used to transport NG from production sites to consumers. Flowlines transport products from the wellhead to the well pad, gathering lines transport NG products from the well pad to processing plants, transmission pipelines transport refined NG across large distances, and distribution lines transport NG products to the consumer. Within this NG transportation system, pipeline damage can occur through corrosion, ground settlement, or excavation damage, resulting in below-ground NG leaks and emission into the atmosphere.

Despite its increased use as a cleaner fuel, NG is both a climate and public safety threat, consisting of between 70 and 90% methane (CH_4_) [3]. Methane is a powerful greenhouse gas (GHG), with 28 times greater global warming potential than CO_2_ over 100 years [4], and is combustible when it collects to a concentration of between 5 and 15% [5]. In 2021, CH_4_ emissions from anthropogenic sources accounted for 12% of the total GHG emissions; of these, 23% came from oil and gas systems [6]. Recent studies suggest that CH_4_ emissions from below-ground NG pipelines are a non-trivial source within the O&G system [7]. Recent studies have reported on leak density and average emissions. Phillips et al. (2020) [8] reported 4.3 leaks per mile of pipeline in Boston, Jackson et al. (2014) [9] reported 3.9 leaks per mile of pipeline in Washington with emissions ranging from 0.3 to 0.9 kg CH_4_ h^−1^, and Weller et al. (2020) [10] estimated a US national average of 0.5 leaks per mile of pipeline emitting 0.12 kg CH_4_ leak^−1^ h^−1^. The US regulates the distribution network through the Department of Transportation’s Pipeline and Hazardous Materials Safety Administration (PHMSA). To mitigate leaks in the distribution network, the PHMSA’s Title 49 of the Code of Federal Regulations, Part 192, sets regulations governing pipeline safety, including regularly conducting pipeline inspections, leak detection, repair, and the reporting of gas leaks [11].

To comply with regulations, NG pipeline operators use hand-held industry-standard instrumentation to detect gas leaks by conducting walking surveys. To conduct a walking survey, an operator walks along a pipeline right of way while screening for above background CH_4_ concentrations. Relatively low-precision instruments such as the Bascom-Turner Gas Rover (Measurement Range: 1 to 10,000 ppm, accuracy: ±2%), and the Remote Methane Leak Detector (RMLD; range: 0 to 99.9999 ppm-m; accuracy: ±5 ppm-m), are used in a walking survey. The Gas Rover draws air from an inlet typically pressed against the ground and measures the mixing ratio of CH_4_ in the air sample in parts per million (ppm) or in percentage of gas, depending on the concentration of gas. The RMLD is a laser-based instrument and reports the line-averaged CH_4_ mixing ratio along the laser path. When surveying a pipeline using an RMLD, the operator points the RMLD’s laser at the suspected leak or across a suspected gas plume 15–30 m away [12]. As these CH_4_ detectors have relatively low precision and resolution, observations made during walking surveys are typically considered a binary indication of a below-ground leak and are not currently used to infer the size of a leak rate.

The quantification of emission rate is more typically carried out during driving surveys [8,9,13]. Driving surveys comprise an inlet mounted on a vehicle between 0.5 and 5 m above ground level (AGL), traveling between 4 and 11 m s^−1^ [13]. The inlet is connected to a CH_4_ analyzer capable of measuring CH_4_ mixing ratios in parts per billion (ppb), such as the Picarro G2301 cavity ringdown spectroscopy gas analyzer (www.picarro.com (accessed on 5 September 2023); measurement range: 0 to 20 ppm; precision: 1 σ over 5 s < 0.5 ppb; accuracy: ±1 ppb over 24 h). The major shortcomings of trace CH_4_ analyzers are that they are typically more expensive than hand-held detectors and require either main power or batteries that only last a few hours. 

The CH_4_ analyzers used in mobile surveys have higher precision and lower detection thresholds than the detectors used in walking surveys; therefore, these can be used to quantify emissions when measured concentrations and meteorological data are used as inputs in an atmospheric dispersion model. However, these instruments require specialist operators and are prohibitively expensive. Lower-cost leak quantification approaches exist but require exposure to the pipeline, such as the Hi-Flow sampler, or multiple measurements using chamber methods. Both methods are detailed further in Supplementary Material Section S1.1 [14,15,16] and share the shortcomings of time, being expensive, and being inconvenient if the leak is in a public area. To date, no unobtrusive method uses available instrumentation to quantify the rate of a sub-surface pipeline leak.

Gas leak classification and repair prioritization are based on above-ground and below-ground gas reading levels and the distance to a nearby building. According to the Gas Piping and Technology Committee [17], a grade 1 leak is a leak that requires immediate repair and continuous assessment to mitigate hazardous conditions. Any reading from inside a building, from an area near a transmission main, and from sub-surface structures (manholes or vaults) within 5 ft of a building that is ≥4% gas in the air is a grade 1 leak [17]. A grade 2 leak is (1) any reading that is <10% gas in the air (within 30 ft of a building) on a paved area, (2) any reading that is ≥30% gas in the air (within 50 ft of a building) on a paved area, (3) any reading of <20% gas in the air (within 20 ft of a building) on an unpaved area, (4) any reading of ≥10% gas in the air (within 30 ft of a building) on a paved area, (5) any reading of ≥20% gas in the air (within 20 ft of a building) on an unpaved area, and (6) any reading that is ≥30% gas in the air (within 50 ft of a building) on an unpaved area. A grade 2 leak should be inspected every 2 months and repaired within 12 months. Any reading that is less than 30% gas in the air within 50 ft of a building is a grade 3 leak.

Under recent guidelines proposed by PHMSA (2023) [18] addressing Section 113 of the PIPES Act of 2020, Part 192, regulated gathering pipelines and transmission pipelines should classify and repair leaks based on the guidelines provided by GPTC. These guidelines prioritize the repair of leaks that pose a risk to both public safety and the environment. However, the prioritization of gas leak classification and repair currently depend on measured concentrations and the location relative to a building or structure. These measured concentrations change with environmental conditions such as wind speed and atmospheric stability [19]. In periods of increased wind speed and low atmospheric stability, enhanced vertical transport within the near-surface atmosphere results in larger emission rates from the surface and a lower observed surface concentration above a leak [19].

Despite NG leaks being both a safety and an environmental concern, there is currently no way to quickly estimate the size of the below-ground leak rate and effectively prioritize leaks for repair. Leak detection methods use instrumentation that reports CH_4_ mixing ratios in ppm but are not currently used for emission quantification. This study investigates whether CH_4_ mixing ratios from an industry-standard instrument can be used to infer a pipeline’s below-ground gas leak rate. Specifically, the objectives of this study are to (1) estimate the surface CH_4_ emission rate using CH_4_ mixing ratios and meteorological data, and thereafter, derive the below-ground leak rate; (2) assess the accuracy of this approach by comparing the estimated leak rates with the controlled release rates under different environmental conditions and atmospheric stabilities; and (3) determine the meteorological conditions and atmospheric stabilities where this approach works best.

To the authors’ knowledge, this is the first study to develop a below-ground leak rate estimation model that uses measurements from common handheld leak detection instruments. This modeling approach intends to extend operators’ capability for leak assessment using instruments they already possess. The novelty of this approach lies in the operator’s ability to detect a leak, and subsequently use above-ground measurements to estimate the leak rate. Ultimately, this approach aims to prioritize the largest below-ground leaks for repair using a flexible leak quantification tool.

## 2. Materials and Methods

The modeling approach used in this study quantifies a below-ground leak rate in three sequential steps: (1) take above-ground downwind measurements of CH_4_ and meteorological data; (2) use the above-ground measurements in a backward Lagrangian stochastic (bLs) atmospheric dispersion model to estimate the surface emission rate; and (3) use the surface-emission rate to calculate the below-ground leak rate via the inverse ESCAPE model (Supplementary Materials, Section S2.1).

### 2.1. Above-Ground Measurements

Experiments were conducted at Colorado State University’s Methane Emissions Technology Evaluation Center (METEC facility) in Fort Collins, CO, USA. The facility consists of testbeds that simulate below-ground NG pipeline leaks using controlled gas releases under varied soil conditions, leak depths, surface cover, and atmospheric weather conditions. Detailed descriptions of the pipeline testbed design can be found in Jayarathne et al. (ref [20]). Gas is supplied to the below-ground release point from compressed NG cylinders (containing 85–95% CH_4_) through a thermal mass flow meter calibrated with known-flow-rate nitrogen. The controlled release rate is obtained as a product of a correction factor (derived from the gas density and coefficient of specific heat) and the flow rate of nitrogen. The flow rate is controlled by solenoid valves and precision orifices. The CH_4_ content of the NG in each controlled release is measured via gas chromatography and accounted for in the known emission rate.

Above-ground measurements were taken from the ‘rural testbed’ at METEC between 27 September and 3 December 2021. The testbed comprised a permeable (grass) surface cover with leak depths of 0.6 m, 0.9 m, and 1.8 m [20]. Gas was supplied through a PTFE (polytetrafluoroethylene) tubing with a diameter of 6.35 mm (model SS-MD-4, Swagelok, Solon, OH, USA) installed at 0.9 m below the ground to simulate a buried pipeline gas leak. The buried end of the tubing was capped with a 3 µm filter, covered using gravel to avoid soil packing, and then, backfilled with local soil.

Controlled release rates were set at 0.2, 0.4, and 0.8 kg h^−1^ (Table 1) for different experiments to investigate small to medium pipeline leaks [15,21,22]. Large distribution leaks greater than 1 kg CH_4_ h^−1^ [15,21,22] were not tested due to concerns about damaging the test bed. Gas was released 24 h before each experiment to establish a steady-state flow between the leak point and the surface. Studies by Mitton (2018) [23] and Tian et al. (2022) [24] show that a steady state can be achieved in approximately 4 h for the selected leak rates. According to Gao et al. (2021) [25], a steady-state gas flow at the testbeds is shown by a negligible change in surface concentration at the point that is situated directly above the below-ground leak point.

**Figure 1:**
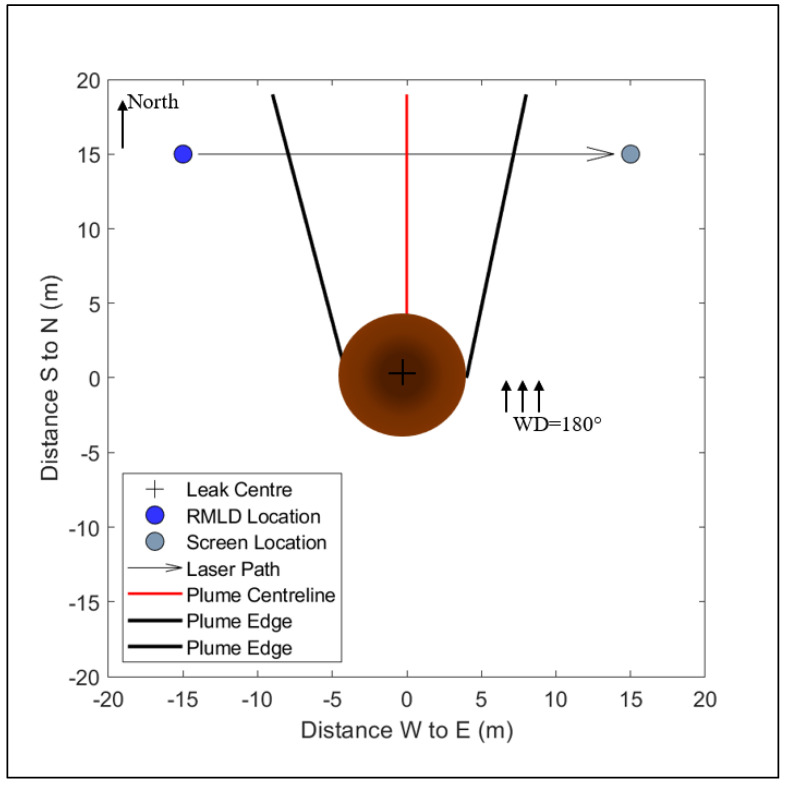
Top-view schematic representation of the surface expression of a leak, location of the RMLD, and the screen during an experiment when the wind direction is 180° (from the south). The emission radius is set at 4 m to represent the distance of surface gas migration from the leak center. The plume centerline has the highest concentrations of CH_4_, and the concentrations decrease towards the plume edge. The above-ground setup was as follows: (i) The reflective screen was located directly opposite the RMLD in all experiments, (ii) the combination was positioned 15 m downwind of the leak, and (iii) the path length was set perpendicular to the wind direction.

All above-ground CH_4_ mixing ratios were measured every 2 s using the intrinsically safe version of the Remote Methane Leak Detector, henceforth referred to as the RMLD (Health Consultants Inc., Houston, TX, USA). The RMLD is an open-path Tunable Diode Laser Absorption Spectrometer (TDLAS) that reports line-averaged CH_4_ mixing ratios in parts per million-meter (ppm-m). The RMLD’s laser unit was mounted at 2 m AGL during an experiment. A reflective screen was set up directly opposite the laser, between 10 and 20 m away. The laser/screen set was positioned at a downwind distance of 10 m from the source. The laser path was set perpendicular to the wind direction (Figure 1). One advantage of using a line-averaging sensor is that it makes it possible to survey a location from a distance [12]; this is useful in areas where the leak location is inaccessible. Heath (2009) [12] recommends measuring at greater than 5 m from the source. Reducing the measurement distance increases the possibility of false detection. This is because the laser beams’ footprint decreases and may not capture an above-ground plume.

Before each measurement period, the RMLD was self-tested [12] (Supplementary Materials Section S3.1). A self-test is performed by removing the controller from the carrying case, turning on the RMLD, and allowing it to warm up for 2 to 3 min. Considering that the RMLD is used by oil and gas operators to detect leaks and is not routinely used for the accurate quantification of leak rates, we used a Picarro G4302 Gas Scouter (Picarro Inc., Santa Clara, CA, USA; Precision: 3 ppb in 1 s) to validate the CH_4_ mixing ratios. For validation (Swagelok, USA, Section S3.2), CH_4_ mixing ratio measurements were sampled side-by-side using both the RMLD and the Picarro.

Meteorological data, including wind speed, wind direction (° East of North), relative humidity, and air temperature, were measured using a sonic anemometer (Model 81000, R. M. Young Co., Traverse City, MI, USA). The sonic anemometer is installed 6 m AGL and located 20 m West and 50 m North of the leak center. Since meteorological data were recorded at 6 m AGL, wind speed was corrected to 2 m to match the measurement height of CH_4_ using Equation (1) [26,27].

(1) $${WS}_{2}={WS}_{6}\times \frac{\ln(\frac{h_{2}-d}{Z_{0}})}{\ln(\frac{h_{6}-d}{Z_{0}})}$$

Wind speed at 2 m AGL (WS_2_, m s^−1^) is calculated as a function of the wind speed at 6 m AGL (WS_6_, m s^−1^), the heights AGL (h_2_ and h_6_, both m), the zero-plane displacement (d, m), and the surface roughness length (z_0_, m) in meters [26,27]. The z_0_ was set at 0.023 m and d at 0.04 m to represent the short grass cover on the testbed [26,27].

### 2.2. Surface Emission above a Leak

The surface emission rates (Q_s_, g m^2^ s^−1^) were calculated using WindTrax’s bLs model [28] (WindTrax Version 2.0). WindTrax is suitable for near-field measurements, i.e., within 1 km of the source, and is accurate when measurements are taken on a flat surface and within an area free of obstructions [28,29,30]. The bLs model simulates the dispersion of pollutants in the atmosphere by tracking individual particles backward in time from a receptor location to their source [29,30]. We use the bLs model because (1) it is suitable for estimating emissions from an area source using above-ground downwind measurements, and (2) it can be applied to below-ground pipelines installed in rural environments, and beneath flat terrain in an area free of obstructions [28].

Inputs to the bLs model included 10 min average CH_4_ mixing ratios, wind speed, and wind direction. The 10 min CH_4_ mixing ratios, wind speed, and wind direction averages were calculated to represent the mean atmospheric state during an experiment [28]. We used a 10 min averaging interval because the bLs model assumes that each measurement is averaged over a specific period of no less than 10 min. Further, shorter averaging periods are less likely to be a representative mean state of the atmosphere [28]. On the other hand, longer averaging periods (>30 min) are likely to be affected by the rapid changes in the surface layer in a diurnal cycle [24,29,30]. Measurements were classified into 4 different PGSCs (Pasquill–Gifford stability classes A, B, C, D) based on the wind speed and daytime solar insolation [31]. PGSCs A, B, C, and D correspond to extremely unstable, moderately unstable, slightly unstable, and neutral atmospheric conditions, respectively. More details on the atmospheric stability classification are provided in Supplementary Materials Section S2.2.

In the bLs model, the input included setting the surface roughness length at 0.023 m to represent the short grass cover on the testbed (Supplementary Materials Section S2.3) and setting the background CH_4_ mixing ratio, measured upwind of the controlled emission. Other parameters required to run the simulation include the Monin–Obukhov length (L, m) (Supplementary Materials Section S2.4), and the friction velocity (u*, m s^−1^). WindTrax calculated these parameters internally based on the wind speed and the PG stability classes. 

The point on the surface that is located directly above the leak will record the highest concentrations of CH_4_; this is referred to as the ‘focus’ [19]. At 0.5 m distance increments from the focus, a leak that is greater than or equal to 0.2 kg h^−1^ forms an emission radius of up to 4.5 m on the surface. The surface CH_4_ concentrations reduce exponentially as the measurement distance from the focus increases [19]. During the experiments, surface CH_4_ measurements taken using a Bascom-Turner gas rover migrated up to a maximum of 4.5 m from the ‘focus’. Therefore, for the conditions tested in this study (0.2, 0.4, and 0.8 kg h^−1^ controlled leak rates, dry soil, grass surface cover, and no underground preferential pathways), we utilized an area source of emission (radius = 4.5 m) on the surface with the highest CH_4_ concentrations at the point that is directly above the leak [19] (leak center in Figure 1).

However, we acknowledge that the bLs model in WindTrax assumes that the area source is homogenous, and that there are no nearby obstructions at the leak center such as buildings and structures.

### 2.3. Uncertainty Analysis

The causes of uncertainty in the modeling approach are the above-ground CH_4_ measurements, the meteorological measurements (wind speed and wind direction), the surface expression of a leak, and the classification of atmospheric stability. We used above-ground CH_4_ measurements and meteorological data from the 0.4 kg h^−1^ controlled release rate to evaluate the uncertainty in the modeling approach (Supplementary Materials Section S4). The uncertainties were determined through a sensitivity analysis of the modeling approach in 3 different PG stability classes (A, B, and C). PGSC D was not used in the sensitivity analysis as it had too few data points (6 points) to be used for comparison.

### 2.4. The ESCAPE^−1^ Model Input

The ESCAPE^−1^ model was derived from the ESCAPE model (Estimating Surface Concentration Above Pipeline Emissions) initially developed by Riddick et al. (2021) [19]. Using a known leak rate, the ESCAPE model was developed to calculate surface CH_4_ enhancement above a below-ground leak. The model employs an analogy based on Ohm’s law to model the resistance of gas flow in both the soil and atmosphere. The surface enhancement is estimated from the surface flux, the background CH_4_ concentration, and the total atmospheric resistance. The ESCAPE model is described in full detail in Supplementary Materials Section S2.1.

Considering that the ESCAPE model estimates surface CH_4_ enhancements, the ESCAPE^−1^ model could be used to estimate the below-ground leak rate of a pipeline. The ESCAPE^−1^ model (Supplementary Materials Section S2.1) is parameterized from the surface emission rate (Q_s_, g s^−1^) derived using the bLs model, the leak depth (m), the wind speed (m s^−1^), the height at which wind speed is measured (in m), the PGSC, and the Monin–Obukhov length, which is derived based on PGSC. Using the inputs, the model calculates the below-ground leak rate in grams per second (g s^−1^).

## 3. Results

Above-ground CH_4_ mixing ratio measurements were taken from the rural testbed at METEC between 27 September and 3 December 2021 and the controlled release rates were set at 0.2, 0.4, and 0.8 kg h^−1^ (Table 1). During the experiments, wind speeds were generally low (<3 m s^−1^) and the atmosphere was generally unstable (Table 1). The average mixing ratios were in the order of 70 to 110 ppm m. From the raw data, it became apparent that the orientation of the RMLD relative to the plume dispersion and wind speed adversely affected the data, leading to the data quality control rules.

### 3.1. Data Quality Control

#### 3.1.1. Wind Direction

Ideally, the RMLD laser’s path should cut across the centerline of an above-ground gas plume [12] (Figure 1). The gas concentration within the plume is heterogeneous and the plume centerline has the highest concentration, i.e., from this line, the concentration of gas reduces outwards [32] (Supplementary Materials Section S6). The bLs model can accurately calculate emissions when the laser is perpendicular to the plume centerline [30,33] (Figure 1). We suggest that rapid changes in wind direction (by 26°) in a 10 min time interval may align the RMLD’s laser along the plume centerline (Supplementary Materials Section S6), i.e., along the region of high CH_4_ concentrations [32], causing the bLs model to overestimate the surface emission and resulting in a subsequent overestimation of below-ground leak rates.

In addition, a shift in the plume centerline can mean that the sensor is measuring at the plume edge (Figure 1 and Figure S14). At the plume edge, gas particles are defined by extreme and less predictable paths [29,30,32,34]. Methane measurements from the plume edge are an average of low concentrations (Supplementary Materials Figure S14); therefore, these CH_4_ measurements underestimate the below-ground leak rate. As a result of these, data were removed when the change in wind direction was ≥26° over the 10 min averaging period.

**Figure 2:**
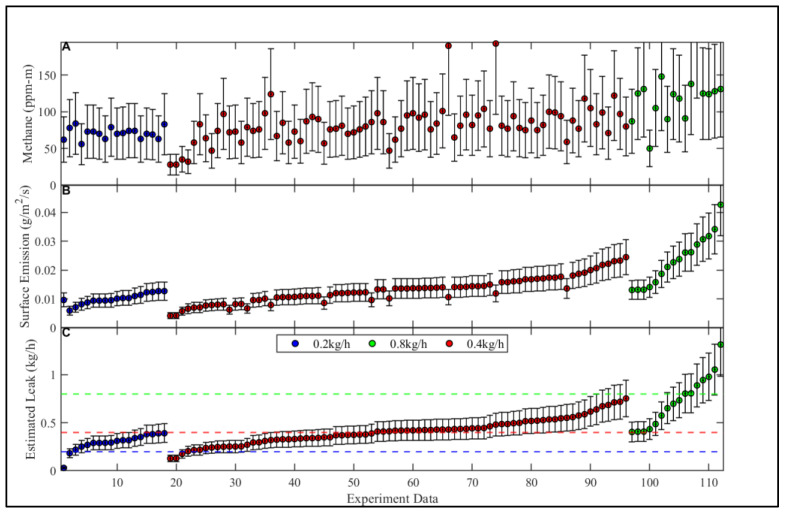
Plot (**A**) shows the 10 min averaged CH_4_ mixing ratios from measurements sampled using the RMLD, plot (**B**) is the surface emission as derived by the bLs model using CH_4_ mixing ratios, and plot (**C**) is the estimated leak rate as derived by the ESCAPE^−1^ model using the surface-emission. The blue, red, and green dotted lines in plot (**C**) are the 0.2, 0.4, and 0.8 kg CH_4_ h^−1^ controlled release rates, respectively. Error bars in plot A represent the uncertainty (±50%) due to the CH_4_ mixing ratios. Error bars in plots (**B**,**C**) represent the uncertainty (±25%) in the modeling approach.

We had a total of 226, 10-min averaged data points (merged CH_4_ measurements, and meteorological data). These data points were classified into PG stability classes A, B, C, and D. Each of the points derived a surface emission rate in the bLs model, and a sequential leak rate in the ESCAPE^−1^ model (Figure 2). After filtering for wind direction, we were left with a total of 173 points.

#### 3.1.2. Wind Speed

Accurate estimation of the surface emission rate and, subsequently, the below-ground leak rate depends on accurate concentration measurement [33]. At low wind speeds, gas may not travel from the source to the RMLD detector, violating the assumptions of constant air flow made by the bLs model [28,33]. The ESCAPE^−1^ model underestimates the leak rate by 54% when the wind speed is less than 2 m s^−1^. Following the experimental methods of Flesch et al. (2004; 2005) [29,30], data were removed when the average wind speed during the experiment was less than 2 m s^−1^. After filtering for wind speed, we were left with a total of 113 data points (Figure 2).

### 3.2. Uncertainty Analysis

The accuracy with which the RMLD could measure the CH_4_ mixing ratio in the air caused the largest uncertainty in leak rate, as calculated using the ESCAPE^−1^ model (±28%). The uncertainties from the wind speed and the wind direction were ±18% and ±8%, respectively. The surface expression of a leak presented an uncertainty of ±38%, whereas the uncertainty in assigning the PGSC using the method described in Supplementary Materials Section S2.2 resulted in an uncertainty of ±30%. We assume that individual uncertainties are inherent. Therefore, we propagated these individual uncertainties to the estimated leak rate to obtain the overall uncertainty (Supplementary Materials Section S4.6). The overall uncertainty in the modeling approach was estimated at ±25% (Table S5).

### 3.3. Methane Mixing Ratios, Surface Emissions, and Calculated Leak Rates

The 10-minute averaged CH_4_ mixing ratios observed during the 113 experiments ranged from 28 ppm m to 193 ppm m. During the 0.2 kg h^−1^ controlled leak experiment, the smallest, largest, and average measured CH_4_ mixing ratios were 56, 84, and 71 ppm-m, respectively (Figure 2A). During the 0.4 kg h^−1^ controlled leak, the smallest, largest, and average measured CH_4_ mixing ratios were 28, 193, and 82 ppm-m, respectively. During the 0.8 kg h^−1^ controlled leak, the smallest, largest, and average measured CH_4_ mixing ratios were 50, 238, and 122 ppm-m, respectively. 

Using the 10-minute averaged CH_4_ mixing ratio in the bLs model, individual surface emissions ranged from 0.010 to 0.043 g m^−2^ s^−1^. For the 0.2 kg h^−1^ controlled leak experiment, the smallest, largest, and average derived surface emission rates were 0.010, 0.013, and 0.010 g m^−2^ s^−1^, respectively (Figure 2B). For the 0.4 kg h^−1^ controlled leak experiment, the smallest, largest, and average derived surface emissions were 0.004, 0.025, and 0.013 g m^−2^ s^−1^, respectively. For the 0.8 kg h^−1^ controlled leak experiment, the smallest, largest, and average derived surface emissions were 0.013, 0.043, and 0.024 g m^−2^ s^−1^, respectively. 

Using the surface emission rates as inputs to the ESCAPE^−1^ model, the individually calculated leak rates ranged from 0.03 to 1.31 kg h^−1^. For the 0.2 kg h^−1^ experiment, the smallest and largest estimated leak rates were 0.03 (−85%) and 0.40 (+100%) kg h^−1^, respectively (Figure 2C). For the 0.4 kg h^−1^ experiment, the smallest and largest estimated leak rates were 0.13 (−68%) and 0.75 (+88%) kg h^−1^, respectively. For the 0.8 kg h^−1^ experiment, the smallest and largest estimated leak rates were 0.40 (−50%) and 1.31 (+64%) kg h^−1^, respectively.

The results of a Pearson’s correlation test show that relative humidity and temperature do not correlate with the estimated leak rate (R^2^ = 0.015 and 0.104, respectively; Supplementary Materials Section S8); as such, we do not focus our discussion on these two variables. Both wind speed and atmospheric stability significantly affect the estimated leak rate (*p*-value less than 0.05); therefore, our discussion is focused on these impacts.

### 3.4. Effect of Time Averaging on Calculated Leak Accuracy

**Figure 3:**
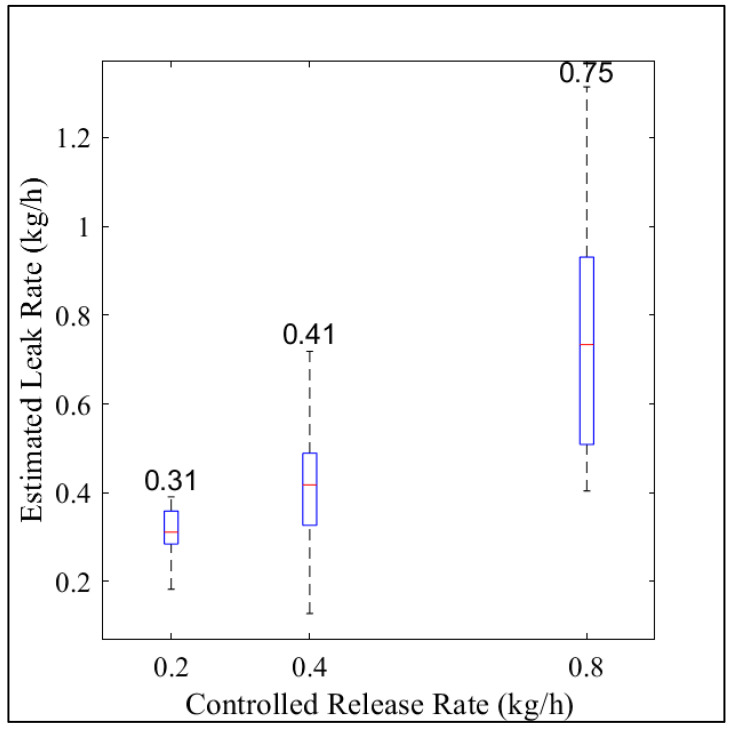
Estimated leak rates from 0.2, 0.4, and 0.8 kg CH_4_ h^−1^ controlled release rates, respectively. The red line inside the box represents the median value, the boxes represent the 25th and the 75th percentiles, and the dotted black lines (the whiskers) represent ±1.5 times the interquartile range. The numbers inside the plot represent the mean estimated leak rate in each controlled release rate. The numbers of data points used are 17, 77, and 15 points in the 0.2, 0.4, and 0.8 kg CH_4_ h^−1^ controlled release rates, respectively.

When all the individual leak rates based on the 10 min averaged data (Figure 2C) were averaged for each known leak rate, the average below-ground leak rates were estimated at 0.31 (95% CI: 0.30, 0.32), 0.41 (95% CI: 0.38, 0.44), and 0.75 (95% CI: 0.69, 0.81) kg CH_4_ h^−1^ for the 0.2, 0.4, and 0.8 kg CH_4_ h^−1^ controlled leaks, respectively (Figure 3). The estimated leak rates in Figure 3 were averaged over 5, 3, and 4 h for the 0.2, 0.4, and 0.8 kg CH_4_ h^−1^ controlled leaks, respectively, as compared to the 10 min averages in Figure 2. These results suggest that the ESCAPE^−1^ model overestimates small leaks (0.2 kg CH_4_ h^−1^) by an average of 0.11kg h^−1^ and underestimates large leaks (0.8 kg CH_4_ h^−1^) by an average of 0.05 kg h^−1^. The linear regression of the average calculated leak rates against the known leak rate shows good agreement (m = 0.75; R^2^ = 0.99, *p*-value = 0.07).

### 3.5. Effect of Wind Speed on the Calculated Leak Accuracy

**Figure 4:**
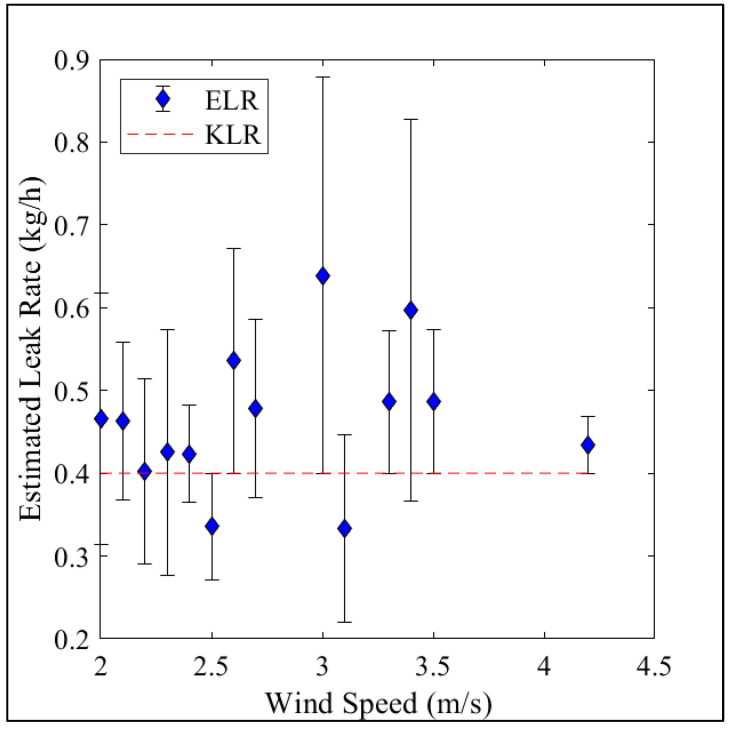
Average estimated leak rate at wind speeds from 2 to 4.5 m s^−1^. The red line is the known leak rate (KLR), and the blue points are the estimated leak rates (ELR). Error bars represent the RMSE from the known leak rate. The model underestimates the leak rate when the wind speed is below 2 m s^−1^. Data from the 0.4 kg CH_4_ h^−1^ controlled leak rate were used to generate this plot.

A Pearson’s correlation test shows that wind speed has a statistically significant impact on the deviation of the estimated leak rate from the known leak rate (m = 0.23, R^2^ = 0.47, *p*-value = 2.88 × 10^−9^). Here, the estimated leak rate is more accurate at lower wind speeds (Figure 4). When the wind speed is between 2 and 3 m s^−1^, the estimated leak rate deviates by 0.1 kg h^−1^ from the known leak rate. As the wind speed increases to 4.5 m s^−1^, the estimated leak rate deviates by 0.2 kg h^−1^ from the known leak rate.

### 3.6. Effect of Atmospheric Stability on Calculated Leak Accuracy

**Figure 5:**
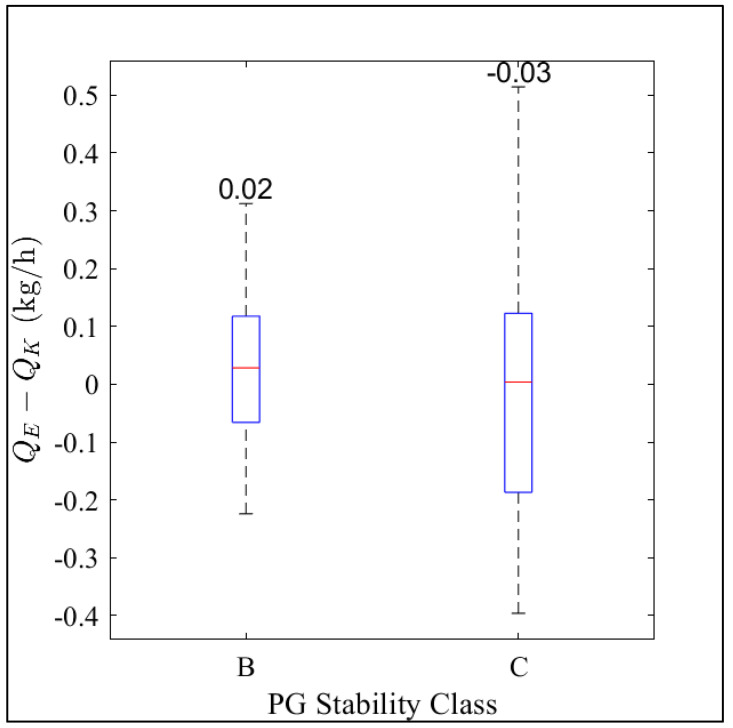
Difference between the estimated and the controlled release rate (Q_E_ − Q_K_) in PGSC B and C. The red lines inside the boxes represent the median value, while the boxes represent the 25th and the 75th percentiles. The dotted black lines (the whiskers) represent ±1.5 times the interquartile range. The numbers inside the plots represent the average difference in each stability class. The numbers of data points used are 73 and 27 in PGSC B and C, respectively. PGSC A was not used in the plot because it has few data points (6) after filtering for WS and changes in WD. Also, PGSC D was not used in the plot because it has 6 data points and represents the neutral conditions of the atmosphere.

Our results show that the ESCAPE^−1^ model estimates the below-ground leak rate with an average bias of 12% and −5% in PGSC B and C, respectively (Figure 5). The difference between the known and estimated leak rates from PGSC A is not shown in Figure 5 because this stability class was filtered out. PGSC A belongs to measurements when the wind speed is less than 2 m s^−1^ and during extremely unstable conditions. This shows that this modeling approach works best during moderately unstable (PGSC B) and during slightly unstable (PGSC C) atmospheric conditions. PG stability class D is not included in the comparison because this category had six data points and represents neutral conditions of the atmosphere.

## 4. Discussion

### 4.1. Instrumentation: The Remote Methane Leak Detector (RMLD)

The work presented here used above-ground, line-averaged CH_4_ mixing ratios measured using a lower-cost, lower-precision TDLAS CH_4_ detector. The results show that when measuring downwind of a below-ground leak point, if the sensor’s laser path cuts across the plume edge, then the reported mixing ratio is likely to be an underestimation of the below-ground leak rate because it will have only measured the edge of the plume, i.e., a region of lower concentrations of CH_4_. This can happen when the wind direction varies more than 25° over the 10 min measurement period or during extremely unstable, low-wind atmospheric conditions when the plume can loft over the laser’s path, i.e., PGSC A. Therefore, we recommend that the below-ground leak rate quantification approach presented here only be used if the laser path is perpendicular to and at the same height as the plume centerline (Figure 1). 

### 4.2. Performance of the ESCAPE^−1^ Model

Using 10 min averaged CH_4_ mixing/meteorological data and filtering out low wind/PGSC A events with a 95% confidence interval, the ESCAPE^−1^ model estimates small distribution leaks (0.2 kg CH_4_ h^−1^) as −31%/75% of the actual leak rate, and medium distribution leaks (0.8 kg CH_4_ h^−1^) as −73%/92% of the actual leak rate (Figure 2C). Large distribution leaks greater than 1 kg CH_4_ h^−1^ [15,21,22] were not tested due to concerns about damaging the test bed. When averaged over a longer period (more than 3 h of data), the average calculated leak rate was an overestimation of +55% for the small (0.2 kg CH_4_ h^−1^) leak and an underestimation of −6% for a medium distribution leak (0.8 kg CH_4_ h^−1^).

These data suggest that the 10 min averaged data used as an input to the ESCAPE^−1^ approach could be used to distinguish between small and medium distribution leaks, but it can more accurately quantify larger leaks than smaller leaks, and its accuracy improves if the measurement time is increased. This is likely caused by the size of the plume created by the emission, where larger leak rates result in a larger plume, and it is more probable that the instrument’s laser path will be perpendicular and level with the plume centerline. The ESCAPE^−1^ model works poorly during extremely unstable atmospheric conditions and at wind speeds less than 2 m s^−1^. If used in these conditions, it will likely underestimate the below-ground leak rate and the total emissions from a pipeline. 

### 4.3. Effect of Wind Speed

Our results also suggest that as the wind speed increases from 2 to 5 m s^−1^, both the accuracy and precision of the leak rate calculated by the ESCAPE^−1^ model decrease. Wind, and particularly its turbulence, is a significant agent in dispersing any gas released into the atmosphere [33]. Therefore, as the wind speed increases, gas disperses less as it travels from the source to the atmosphere, resulting in a collimated plume that could be undetected by the instrument’s laser.

Observations at the METEC site suggest that wind speeds are often less than 2 m s^−1^ (Supplementary Materials Section S7), and studies have shown that most atmospheric dispersion models, including the bLs model, are inaccurate in estimating emissions during low wind speeds [30,34,35,36]. In the real world, waiting for ideal wind conditions to estimate emissions may conflict with the operator’s schedule, and measurements need to be made regardless of meteorology.

### 4.4. Effect of Atmospheric Stability

In addition to wind speed, our results also indicate that the ESCAPE^−1^ model is better at estimating the below-ground leak rate as the atmosphere becomes more unstable, i.e., PGSC B is better than PGSC C. As mentioned above, PGSC A data should not be used as it is likely that the plume will not reach the detector in low wind and an extremely unstable atmosphere. We suggest that PGSC B conditions result in better leak size estimates as the plume will be more vertically mixed with a less defined plume center that could be undetected by the laser. This may be a result of the long path length of the RMLD, which accounts for the effects of atmospheric turbulence, but also suggests something of a measurement pay-off between atmospheric conditions that transport the gas from the source to the detector, but also with enough vertical mixing to make the plume more homogenous.

### 4.5. Suggested Improvements to the Modeling Approach

The main sources of uncertainty in the modeling approach were the measurement of the CH_4_ mixing ratios using a line-averaging TDLAS detector and atmospheric stability classification. We picked a line-averaged TDLAS instead of a point sensor, i.e., the Gas Rover, for above-ground CH_4_ measurements because it provides flexibility in accessing difficult-to-access leak locations and would measure across the plume, i.e., is more likely to detect the plume. However, the accuracy in the TDLAS instrument depends on how well the gas has dispersed, allowing the laser to cross the plume centerline. As described in Section 4.4, we suggest that the ‘better measurement condition’ resulting in more accurate quantification using the ESCAPE^−1^ approach is something of a pay-off between wind being high enough to move the gas, but not so high that the plume becomes collimated and more easily undetected. 

Atmospheric stability can be classified in many ways, including using Pasquill–Gifford’s classification [31] or the Monin–Obukhov length [29,37]. Here, we elected to use the relatively low-tech approach of estimating the PGSC using wind speed and isolation as it is unlikely that operators would have access to instruments that could quantify the Monin–Obukhov length. As our uncertainty analysis has shown, the uncertainty associated with assigning PGSC is ±30%, but this could be reduced by using a sonic anemometer, where the Monin–Obukhov length can be directly calculated, or by measuring wind speed and different heights and inferring the Monin–Obukhov length from the change in wind concerning height above the ground [37].

As the RMLD can respond faster than the 10 min average used here, a shorter measurement time could be employed by the operator. However, as shown above, an increase in measurement time decreases the associated leak quantification uncertainty. This suggests that if a shorter measurement period was used, the calculated leak rate would be less representative of the actual leak rate. Here, we suggest that a 10 min average is a good compromise between accuracy and utility.

### 4.6. Measuring in More Complex Environments

The approach presented here was tested on a rural testbed comprising a grass surface with no sub-surface infrastructure; this only simulates a pipeline passing through undisturbed homogeneous soil. For gathering and transmission lines, this may be reflective of the situation, but is unlikely to simulate what is happening in the distribution network. All the measurements were taken on flat terrain with a vegetation surface cover (surface roughness = 2.3 cm for short grass: Supplementary Materials Table S3) and minimal obstruction such as nearby buildings (Supplementary Materials Figure S2). Here, we suggest that the ESCAPE^−1^ model may not work to estimate pipeline leaks in complex aerodynamic environments such as an urban landscape. To address this, we suggest testing the approach in more aerodynamically complex environments, where measurements are taken in an area with obstructions such as tall buildings, and above a less permeable surface cover such as asphalt. This will create a clear understanding of surface and above-ground gas flow in less permeable surface cover and possibly improve gas leak quantification in these complex geometries.

## 5. Conclusions

This study investigated the environmental conditions in which lower-cost, industry-standard CH_4_ detectors could be used to quantify leak rates from subsurface NG pipeline leaks installed in rural environments. Here, we show that averaged CH_4_ concentration data measured using a CH_4_ detector can be used to calculate leak rates between 0.2 and 0.8 kg CH_4_ h^−1^ at wind speeds above 2 m s^−1^ when the pipeline is traveling through native soils. At minimum, this tool can be used to differentiate between a small (0.2 kg CH_4_ h^−1^) and a medium leak (0.8 kg CH_4_ h^−1^); this makes it possible to prioritize leak repair, as current methods only use CH_4_ detector data to generate a binary decision on whether the leak is a safety risk or not. 

Recently, the PHMSA proposed a revision to the regulations that implement the PIPES Act of 2020. The amendments would include modernizing old pipeline leak detection, leak grading, and leak repair rules by using technological methods to detect, grade, and repair leaks [18]. Current methods such as flux chambers involve exposing the pipeline to accurately quantify the leak rate. Emission from a below-ground leak covers a large area on the surface, and digging such an area to expose a pipeline takes time, yet operators cannot pinpoint the exact point in the pipeline that is leaking before exposing it. Our modeling approach provides a quick and efficient way of quantifying a below-ground leak while eliminating the need to expose a pipeline. Particularly, this may be of value when estimating leak size in the gathering pipeline network. 

In line with modernizing traditional methods in pipeline safety, the modeling approach detailed in this study leverages industry-standard instruments to estimate the below-ground leak rate. The RMLD is a path-integrated instrument that can detect a leak from up to 30 m away; this means that it is suitable for leak locations that are ‘hard-to-reach’. Once an operator localizes a leak using the RMLD, they can use above-ground measurements to estimate the leak rate in less than 30 min without digging up the pipeline. This is suggested because it takes 10 min to take above-ground measurements, a maximum of 5 min to estimate surface emission, and a maximum of 5 min to estimate the leak rate. In less than 30 min, this approach can estimate the leak rate with an accuracy of 75%, obtained as a difference between a 100% ideal accuracy and the 25% uncertainty of the modeling approach. 

Future work could investigate whether this approach could be applied to distribution pipeline networks laid in both relatively simple terrain and topographically complex environments, with the potential of simulating multiple leak points in the distribution network.

## Figures and Tables

**Table 1:** Experiments were conducted at Colorado State University’s METEC facility to test the performance and accuracy of the modeling approach in estimating below-ground leak rates. The table shows the controlled release rates, duration of the experiments (in hours), CH_4_ mixing ratios (averaged over the duration), wind speed (in m s^−1^), and PGSC (Pasquill–Gifford Stability Class).

Experiment No:	Controlled Release Rate (kg h^−1^)	Duration (h)	CH_4_ Mixing Ratios (ppm-m)	Wind Speed (m s^−1^)	PGSC
1	0.2	6	73	2.6	B
2	0.4	6	83	1.3	B
3	0.4	3	89	3.4	D
4	0.4	6	89	2.1	B
5	0.4	4	85	1.2	A
6	0.4	5	75	1.8	A
7	0.8	4	73	1.0	A
8	0.8	3	109	2.0	C

## Data Availability

The data sets for this study are found in the reference: Fancy Cheptonui; Riddick N. Stuart; Anna Hodshire; Mercy Mbua; Kathleen M. Smits; Daniel J. Zimmerle; Replication Data for Estimating the below-ground leak rate of a Natural Gas pipeline using above-ground downwind measurements: THE ESCAPE^−1^ MODEL, https://datadryad.org/stash/share/hrNNi7QftejUvLLT2Nahi2tM26ilVLk36_Qe8NYAToM (accessed on 6 September 2023), Dryad data repository.

## Supplementary Materials

The following supporting information can be downloaded at: https://www.mdpi.com/article/10.3390/s23208417/s1. Figure S1: Below-ground to above-ground gas flow showing the assumptions made by the ESCAPE model; Figure S2: The RMLD setup during calibration and during an experiment; Figure S3: Methane measurements from the RMLD versus Methane measurements from the Picarro; Figure S4: Representation of the setup of the RMLD and Picarro during calibration; Figure S5: Slopes obtained from bootstrap resampling (95% confidence interval: 0.43 to 0.74); Figures S6–S9: The estimated leak rates from, the RMLD accuracy of 28%, the varied wind speeds, the varied wind direction, and the varied radius of the source in PG stability classes A, B, and C, respectively; Figures S10–S13: A linear relationship between the estimated leak rate and, the Methane mixing ratios, the wind speed, the wind direction, and the radius of the source, respectively; Figure S14: Dispersion of gas in the atmosphere from the plume ‘centerline’; Figure S15: Wind speed rose for the METEC facility between September and December 2021; Table S1: Parameters used in Fick’s and Darcy’s law when applied to the ESCAPE^−1^ model; Table S2: Classification of atmospheric stability using the Pasquill Gifford Stability Classes (PGSC) based on wind speed (m s^−1^) and daytime insolation; Table S3: The roughness length of different surfaces is based on the vegetation cover of the site; Table S4: Table for estimating the Monin-Obukhov length (L, m) based on the Pasquill-Gifford Stability class (PGSC); Table S5: Derivatives and standard deviation of each cause of uncertainty in the modeling approach; Table S6: Results of a Pearson’s correlation between the estimated leak rate, temperature, and relative humidity.
