# Supplementary material for: Estimating the Below-Ground Leak Rate of a Natural Gas Pipeline Using Above-Ground Downwind Measurements: The ESCAPE−1 Model
Source: Sensors (Basel). 2023 Oct 12;23(20):8417. doi: 10.3390/s23208417 (PMC10610919; doi:10.3390/s23208417)
Supplement: Supplementary file 1 [file sensors-23-08417-s001.zip › sensors-2625370-supplementary.pdf]

Supplementary Information for Estimating the Belowground Leak Rate of a Natural Gas Pipeline using Aboveground Downwind Measurements: THE ESCAPE^{-1} MODEL

Fancy Cheptonui[1,2*], Stuart N. Riddick[1,2], Anna L. Hodshire[2], Mercy Mbua[1,2], Kathleen M. Smits[3], and Daniel J. Zimmerle[2]

[1]Department of Systems Engineering, Colorado State University, Fort Collins CO 80523, USA

[2]Energy Institute, Colorado State University, Fort Collins CO 80524, USA

[3]Department of Civil and Environmental Engineering, Southern Methodist University, Dallas, Texas 75205, USA

Supplementary Materials Section 1

S1.1: Flux chambers and Hi-flow sampler

Flux chambers are classified into two static and dynamic flux chambers. A flux chamber consists of an enclosure placed over the source of emission; for a static chamber, samples are drawn from the chamber at specific time intervals, every 5, 10, and 20 minutes. In a dynamic chamber, samples are taken from the chamber once a steady state is reached and maintained for approximately 10 minutes. According to Riddick et al., (2019), it takes from 1 to 3 hours for a steady state to be reached and maintained for 10 minutes in a dynamic chamber. A gas chromatograph (GC) is used to measure the CH_4 concentrations in the samples from both the static and the dynamic chambers. The emission rate is calculated from the linear increase in CH_4 concentration over time (in a static chamber), or the steady-state (in a dynamic chamber) CH_4 concentrations multiplied by the flow rate of air that is passed through the chamber. Chamber methods are time-consuming, as either the entire emission area must be enclosed by the chamber, or the leaking pipeline exposed to cover the leak.

A high-flow sampler is equipped with a surface enclosure, a catalytic and thermal conductivity hydrocarbon sensor, and a thermal gas flow sensor. When estimating the surface emission, the surface enclosure covers the emission area, the catalytic and thermal conductivity sensor measures CH_4 concentrations in the air stream, and the thermal gas flow sensor measures the sample flow rate. The emission rate is derived by multiplying the CH_4 concentrations by the sample flow rate.

Supplementary Materials Section 2: Modeling Approach

S2.1: The ESCAPE-1 Model

The ESCAPE model was developed by Riddick et al., (2021) to estimate surface CH_4 concentrations. In Equation S1, gas flow to the atmosphere, Q_x, is a function of the gas mixing ratio from the leak (X_l), the gas mixing ratio in the atmosphere (X_{at}), the resistance to gas flow in the soil (R_s), and the resistance to gas flow in the atmosphere (R_{at}).

$$Q_x = \frac{X_l - X_{at}}{R_{at} + R_s} \qquad \text{(S1)}$$

For gas leaks in the gathering and distribution system, the flow in the soil is limited by diffusion, assuming that advection affects only a small section near the leak. Here, the rate of diffusion is a function of the concentration gradient and the diffusion coefficient. Therefore, the diffusive flux in the soil (f_d: Equation S2) is obtained through Fick's law, and is considered a product of the concentration gradient (∇C, g m^{-4}) and diffusion coefficient (D_p, m^2 s^{-1})

$$f_d = -D_p \nabla C \qquad \text{(S2)}$$

To model gas flow in the sub-surface, ESCAPE assumes that:

1. The soil properties affecting diffusion are uniform in the region.
2. Gas flow is inversely proportional to the resistance of flow.
3. The resistance of gas flow is inversely proportional to the distance the gas will travel to the surface, as affected by soil resistance.
4. Surface emission at a perpendicular distance from the leak can be scaled to the actual size of the leak (Figure S1).
5. Gas flow is in a steady state, and only vents to the atmosphere, i.e., it does not vent into any nearby structures.
6. The atmosphere is homogenous while the soil is not, and there are various pore channels in real soils wherein gas diffuses directly through the channels to the surface.

Using the assumptions above, the ESCAPE model defines the surface CH_4 flux at a distance x from the focus (F_x, g m^{-2} s^{-1}: Equation S3).

$$F_x = \left(\frac{Q_T}{A_x}\right)\left(\frac{Q_x}{\sum_{x=0}^{\infty} Q_x}\right) \qquad \text{(S3)}$$

The surface CH_4 flux is defined as a function of the total emission rate (Q_T), area of the surface expression of the leak (A_x, m^2: Equation S4), emission at a distance x (Q_x, g s^{-1}: Equation S5), and the sum of emission rates at distances from the focus ($\sum Q_x$, g s^{-1}).

$$A_x = \pi\left(\left(x + \frac{x}{2}\right)^2 - \left(x - \frac{x}{2}\right)^2\right) \qquad \text{(S4)}$$

$$Q_x = \frac{V}{\sqrt{X^2 + d^2}} \qquad \text{(S5)}$$

In Equation S5, V is considered the sum of the advective flux, (F_{Dx}, Equation S6), and the diffusive flux (F_d: Table S1).

Substituting for V:

$$Q_x = \frac{F_{Dx} + F_d}{\sqrt{X^2 + d^2}}$$

Where F_{Dx} is given by

$$F_{Dx} = -\frac{kk_r\rho}{\mu}(\nabla P - \rho g) \quad (S6)$$

The data used in Darcy's (Equation S6) and Fick's law can be found in Table S1.

Table S1 Parameters used in Fick's and Darcy's law when applied to the ESCAPE^{-1} model.

Parameter	Value
F_d	0.00185m^3 s^{-1}
K	1 x 10^{-8} m s^{-1}
K_r	0.2
g	9.8 m s^{-2}
μ	1.09 x 10^{-6} m^2 s^{-1}

The diffusive flux describes the flow of gas from the belowground leak to the region surrounding the leak, while advection flux describes the flow of gas at regions further from the belowground leak point. Due to the configuration of the ESCAPE^{-1} model, V is taken as a constant that cancels out when the leak rate is calculated. Even though this was unintentional, this is a bonus, as the ESCAPE^{-1} model was designed to be used by first responders (e.g., firefighters, and pipeline operators) who would not have access to instrumentation capable of measuring the parameters to explicitly quantify the advective and diffusive fluxes.

The resistance to gas flow in the atmosphere (Equation S7) is a sum of the boundary layer resistance (R_b, m s^{-1}: Equation S8) and the aerodynamic resistance (R_a, m s^{-1}: Equation S9).

$$R_{at} = R_a + R_b \quad (S7)$$

The boundary layer resistance describes the flow of gas through the vegetation on the surface (Equation S8). It is a product of the boundary layer Stanton number (B=5) and the friction velocity (u^*, m s^{-1}).

$$R_b = (Bu^*)^{-1} \quad (S8)$$

The aerodynamic resistance (Equation S9) is derived as a function of the zero-plane displacement length (z_d, m), the roughness length (z_0, m), the Monin–Obukhov length (L, m), and the product of the Von Karman constant (k = 0.41) and the wind speed (u, m s^{-1}).

$$R_a = \frac{\left[\ln\left(\frac{z-z_d}{z_0}\right) - \psi_m\left(\frac{z-z_d}{L}\right)\right]^2}{k^2 u} \quad (S9)$$

In Equation S9 above, a stability correction factor (ψ_m) is obtained for both the stable and the unstable conditions (Equations S9.1 and S9.2).

$$\psi_m\left(\frac{z}{L}\right) = -\frac{5z}{L} \quad (S9.1)$$

$$\psi_m\left(\frac{z}{L}\right) = 2\ln\left(\frac{1+X}{2}\right) + \ln\left(\frac{1+X^2}{2}\right) - 2tan^{-1}(X) + \frac{\pi}{2} \quad (S9.2)$$

$$\text{Where, } X = (1 - 16(\tfrac{z}{L}))^{1/4}$$

Applying Ohm's law resistance analogy, the ESCAPE model estimates the surface CH_4 concentration (X_x, ppm: Equation S10) using F_x (Equation S3), a background concentration (X_a) of 1.9 ppm, and atmospheric resistance R_{at} (Equation S7).

$$X_x = F_x R_{at} + X_a \qquad \text{(S10)}$$

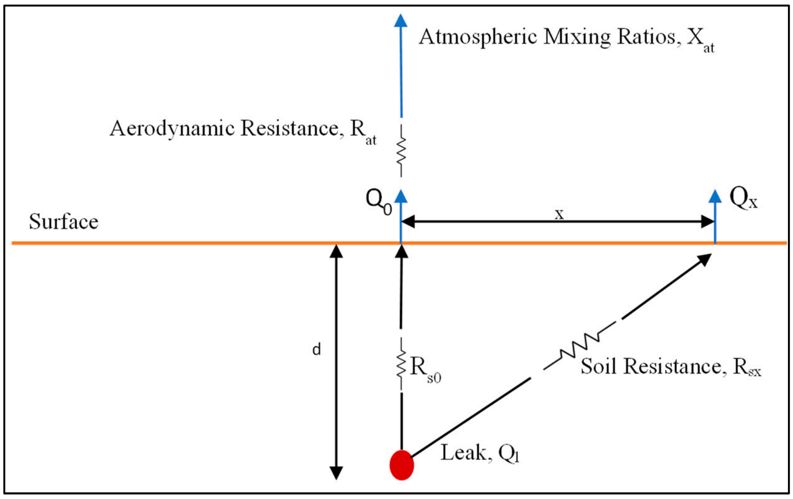

***Figure S1** Belowground to aboveground gas flow showing the assumptions made by the ESCAPE model (Riddick* et al., *2021)*

To estimate the belowground leak rate in the ESCAPE[1] model; the model assumes that the surface emission is the sum of the product of surface fluxes (Q_X: Equation S5), and the area of surface expression (A_X: Equation S4) of the leak ($\sum Q_x A_x$, g m^2 s^{-1}). Here, the model shows that at the surface, the leak presents as an area source (concentric-like gas migration) with the highest concentration of gas at the point that is directly above the leak, known as the leak center in this study.
The emission (Q_x, g s^{-1}) is derived at perpendicular distances from the leak (Figure S1), while the area of the surface expression (A_x: m^2) of the leak at 0.5 m from the focus is derived as a function of the emission radius (Equation S4). Using the surface concentrations at each distance from the focal point, the model estimates individual flow rates from the leak point to each distance on the surface (Q_x, g s^{-1}).
The belowground leak rate (Q_l, g s^{-1}: Equation S11) is derived as a function of an area correction factor (A_f = 0.044 m^2), the emission rates derived by the bLs model (Q_S, g s^{-1}), and the total surface flux ($\sum Q_x A_x$, g m^2 s^{-1}). The area correction factor accounts for the radial presentation of the leak.

$$Q_l = \frac{A_f * Q_S}{\sum_{x=0}^{\infty} Q_x A_x} \qquad \text{(S11)}$$

S2.2: Classification of Atmospheric Stability

Atmospheric stability is the tendency of the atmosphere to resist vertical motion. There are several ways of classifying atmospheric stability. Using Pasquill's measure of atmospheric stability, measurements in this study were classified based on wind speed and daytime insolation. Strong insolation represents sunny midday during midsummer in England, while slight insolation corresponds to similar conditions in midwinter. Measurements in this study were taken during the day (between 10 am to 6 pm); therefore, the stability classes were considered based on daytime insolation. The wind speed during the experiments ranged from 0.7 to 5 m s^{-1}; hence, the data points were classified into PGSC A, B, C, and D (Tables S2).

Table S2. Classification of atmospheric stability using the Pasquill Gifford Stability Classes (PGSC) based on wind speed (m s^{-1}) and daytime insolation. Strong insolation represents sunny midday during midsummer in England, while moderate insolation corresponds to similar conditions in midwinter.

Stability class	Day			Night	
Wind speed (m s^{-1})	Strong	Mod	Light	Overcast	Clear
<2	A	A	B		
3	B	B	C	E	F
4	B	C	C	D	E
5	C	C	D	D	D
>6	C	D	D	D	D

S2.3: Surface Roughness Length

The surface layer model in WindTrax requires the surface roughness length (z_0, m), as one of the parameters used to define the wind profile in a simulation. The roughness length is estimated based on the vegetation cover (Table S3). The rural testbed used in this study is covered by short grass; therefore, a roughness length of 0.023 m was set during each simulation.

Table S3. The roughness length of different surfaces is based on the vegetation cover of the site.

Surface Cover	Roughness Length, m
Sand	0.0003
Bare soil	0.03
Short grass	0.023
Tallgrass	0.1
Wheat	0.15

S2.4: Monin–Obukhov Length

When calculating a belowground leak rate, the Monin–Obukhov length (L, m), a measure of atmospheric stability, it is estimated from the PGSC table (Tables S4).

Table S4. Table for estimating the Monin–Obukhov length (L, m) based on the Pasquill–Gifford Stability class (PGSC)

PGSC	L(m)
A	-5.5
B	-11
C	-30
D	1E+17
E	30
F	11
G	5

Supplementary Material Section 3: Instrument Calibration

The Remote Methane Leak Detector (RMLD; Heath Consultants Inc, Texas, USA) is an instrument that is often used by oil and gas company operators to screen and detect gas leaks. For this reason, the RMLD was calibrated alongside the Picarro G4302 gas analyzer (Picarro Inc, California USA) to validate the measurements. It has an operating range of 0 to 99, 999 ppm m and a sensitivity of 10 ppm m at 30 m (maximum path length). Further, before each experiment, the RMLD was self-tested (Section S3.1).

S3.1: RMLD Self-test

The self-test is based on the procedure outlined by Heath Consultants Inc. First, the controller is removed from the carrying case, and the trans receiver is left inside the carrying case. The RMLD is turned on and allowed to warm for 2 to 3 minutes. From the controller menu, the SELECT button is used to scroll for the SELF-TEST icon. To initiate the self-test, the UP button on the menu is pressed. If the RMLD passes the self-test, the number 255 is displayed on the screen.

S3.2: RMLD Calibration

The RMLD was calibrated by running it alongside the Picarro gas analyzer for 1.3 hours (Figure S2). We used the Picarro gas analyzer because it is a high-precision instrument (precision < 0.5 ppb). Gas was released at 10 standard liters per minute. The RMLD is an open-path Tunable Diode Laser (TDLAS) that averages CH_4 concentrations along the line of sight of the laser path. It reports CH_4 mixing ratios (in parts per million/meter) as a product of the average concentration, and the path length.

During calibration, the trans receiver was placed 2 m (path length) away from the source of the leak, while the inlet of the Picarro was placed 0.5 m above the source (Figure S2). Measurements from the RMLD were converted to parts per million by dividing the mixing ratio by 2 m path length, allowing for comparison with Piccaro measurements. One-minute averages of the data were obtained and used to derive the relationship between the two instruments.

From the result, the RMLD is not sensitive to low CH_4 concentrations (less than 5 ppm/m) as compared to the Picarro (Figure S3). When the values of CH_4 concentrations from the RMLD are plotted against values from the Picarro, a linear relationship is obtained, with an R^2 = 0.65 and m=0.6227 (Figure S3). Measurements from the RMLD are divided by 0.62 as a correction factor.

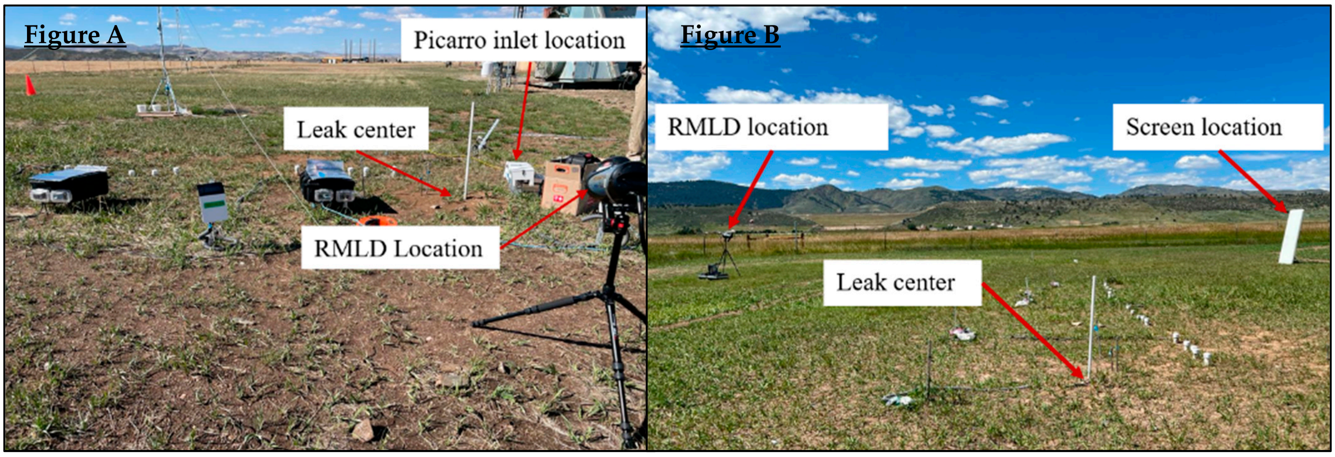

Figure S2 The RMLD setup during calibration and an experiment. Figure A on the left pane represents the setup of the RMLD during calibration with a Picarro gas analyzer, and Figure B on the right pane shows the setup of the RMLD and the reflective screen during aboveground downwind measurement. The diagrammatic representation shown in Figure B above is presented in Figure 1 of the main paper.

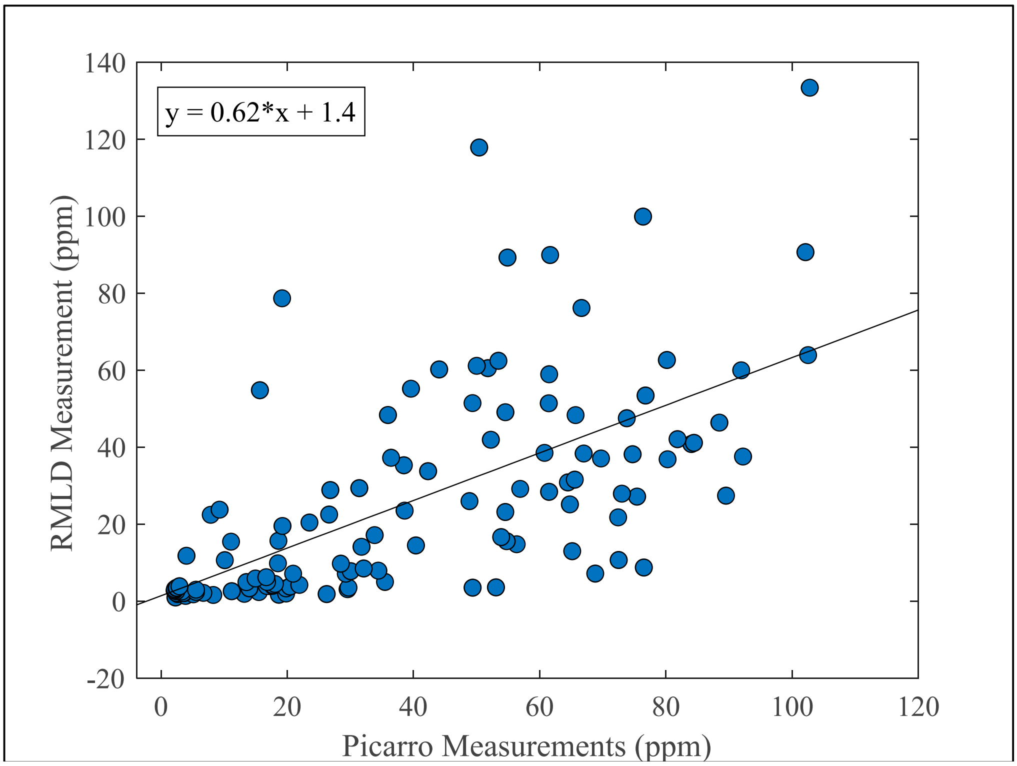

Figure S3 Methane measurements from the RMLD versus methane measurements from the Picarro. The plot shows a linear relationship between the RMLD measurements and the Picarro gas analyzer measurements.

Supplementary Material Section 4: Uncertainty Analysis

The causes of uncertainty in this modeling approach are line-averaged CH_4 mixing ratios measured by the RMLD, wind speed measurement, wind direction measurement, classification of atmospheric stability, and estimating the area of the surface expression of a leak.

The uncertainties are evaluated by performing a sensitivity analysis of the modeling approach in PG stability classes A, B, and C. We used 10-minute-averaged CH_4 mixing ratios and meteorological data from the 0.4 kg h^{-1} controlled leak rate to perform the sensitivity analysis. We assume that the individual uncertainties are inherent in the estimated leak rate; therefore, we perform an uncertainty propagation to combine the uncertainties (Section S4.6).

The major steps in the sensitivity analysis are as follows: (1) to derive the surface emission using WindTrax's bLs model; (2) to derive the belowground leak rate using the ESCAPE-1 model; and (3) to calculate the standard deviation (we use this to present uncertainty in each case) using the estimated leak rate in PG stability classes A, B, and C. The calculated uncertainty from PG stability class B is used to present the uncertainty in the estimated leak rate (0.4kg h^{-1} ± standard deviation in kg h^{-1}).

S4.1: Uncertainty due to line-averaged CH_4 mixing ratios.

The RMLD (measurement range: 0 to 99,9999 ppm-m, sensitivity: ± 5 ppm-m) is sold as a leak detection instrument, and as such does not come with an estimate of the accuracy in its measurements of the CH_4 mixing ratio. To account for this, the RMLD was calibrated by running it alongside the Picarro gas analyzer (Section S3.2).

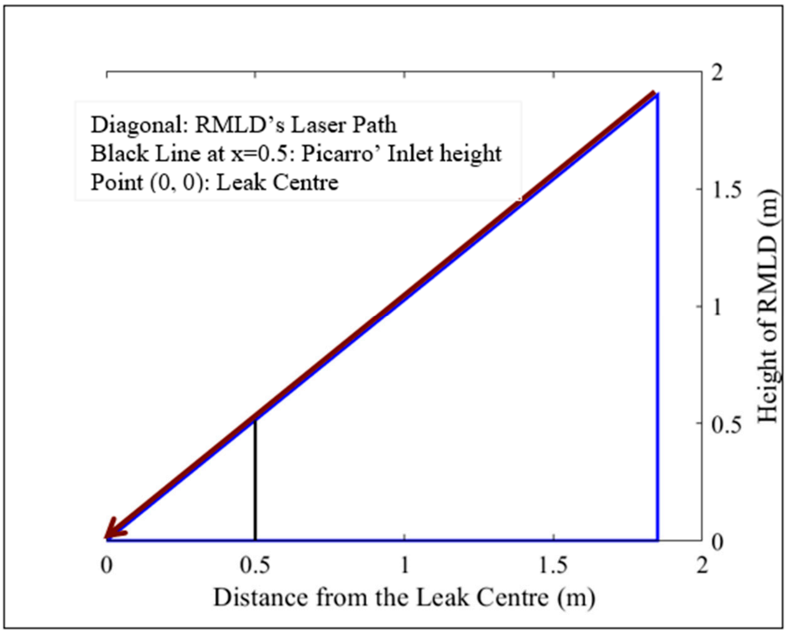

Figure S4: Representation of the setup of the RMLD and Picarro during calibration. The RMLD was positioned such that the laser was located 2 m from the leak center. The Picarro was positioned 0.5m from the leak center. Methane readings were transmitted every 2 seconds in both instruments.

To accurately quantify the accuracy of the RMLD, we employed a Monte Carlo bootstrap fit. A bootstrap fit involved randomly sampling RMLD measurements and Picarro measurements, with replacement to create multiple bootstrap samples. A total of 1000 bootstrap samples were used, and for each sample, a linear regression model was fitted. From this, a 95% confidence interval of the slopes was calculated (Figure S5)

The lower bound and upper bound of the confidence interval were determined using the 2.5th and 97.5th percentiles of the bootstrap slopes, respectively (Figure S5). The mean, upper, and lower bound of the slopes from this sampling are 0.58, 0.74, and 0.45, respectively.

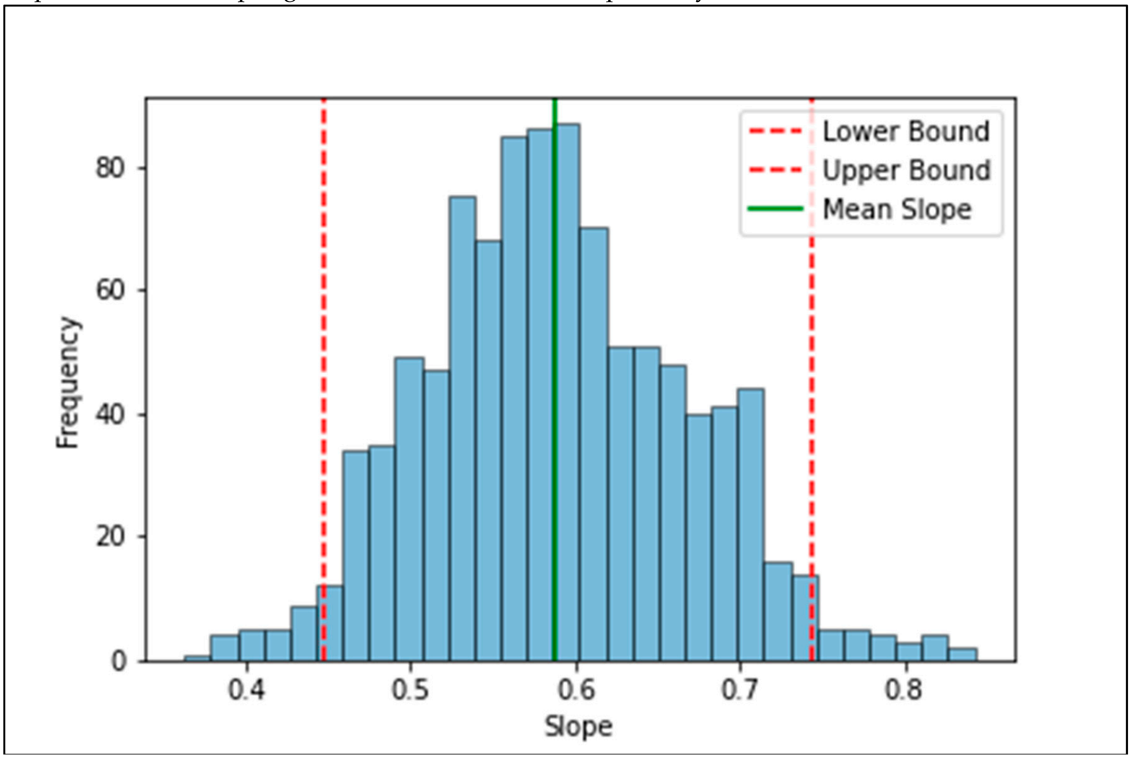

Figure S5: Slopes obtained from bootstrap resampling (95% confidence interval: 0.43 to 0.74). Bootstrap resampling involved sampling RMLD measurements and the Picarro measurements, using replacements to create multiple linear regression models. A slope for each sample was then calculated. A 95 % confidence interval was derived by using slopes between the 2.5th to 97.5th percentiles. A figure of 0.45 represents the slope in the 2.5th percentile, while 0.74 represents the 97.5th percentile.

Based on the analysis, the accuracy of the RMLD was derived using the mean, and the upper bound from the bootstrap fit (Equation S12). The accuracy is ±28%

$$R_U = \frac{Upper\ bound - Mean}{Mean} \quad \text{(S12)}$$

The uncertainty associated with the CH_4 mixing ratios was evaluated using an RMLD accuracy of $\pm28\%$. The upper and lower bounds of the CH_4 mixing ratios were obtained as $M_i \pm (0.28M_i)$, where M_i is the CH_4 mixing ratio. The input to WindTrax included varied CH_4 mixing ratios, varied stability classes, a constant area of the source, constant wind direction, constant wind speed, and constant downwind position of the RMLD. The surface emission rates were calculated in WindTrax and used to calculate subsurface leak rates in the ESCAPE-1 model. A standard deviation was obtained for each PG stability class as $\sqrt{\frac{\sum_i^n (ELR_i - 0.4)^2}{(n-1)}}$, where ELR is the estimated leak rate, and n is the number of data points in a stability class. Based on PG stability class B, the uncertainty associated with CH_4 mixing ratios is (0.40±0.20kg/h).

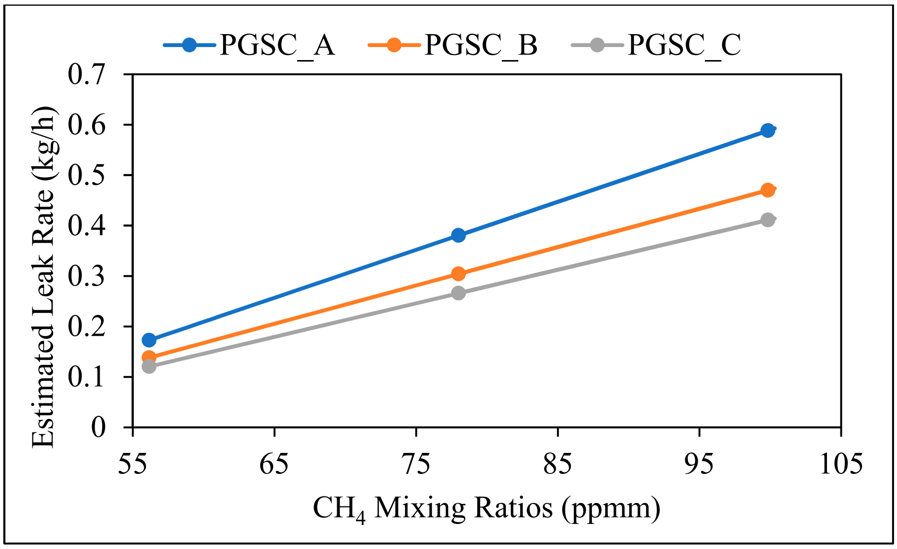

Figure S6: The estimated leak rates based on the RMLD accuracy of 28% in PG stability classes A, B, and C.

S4.2: Uncertainty due to wind speed measurement

To derive the surface emission, wind speed measurement is used as one of the inputs to WindTrax. Therefore, to assess the impact this might have on the estimated leak rate, a sensitivity analysis was carried out. The uncertainty associated with the wind speed measurements was evaluated using a sonic anemometer accuracy of $\pm 1\%$. The upper and lower bounds of the wind speed were obtained as $WS_i \pm (0.01WS_i)$, where WS_i is the wind speed measurement. The input to WindTrax includes varied wind speed, varied PGSC, a constant area of the source, constant wind direction, constant CH_4 mixing ratios, and constant downwind position of the RMLD. The surface emission rates were calculated in WindTrax and used to calculate the subsurface leak rates in the ESCAPE^{-1} model. A standard deviation was obtained for each PG stability class as $\sqrt{\frac{\sum_i^n (ELR_i - 0.4)^2}{(n-1)}}$, where ELR is the estimated leak rate, and n is the number of data points in a stability class. Based on PG stability class B, the uncertainty associated with wind speed is (0.4±0.07kg/h).

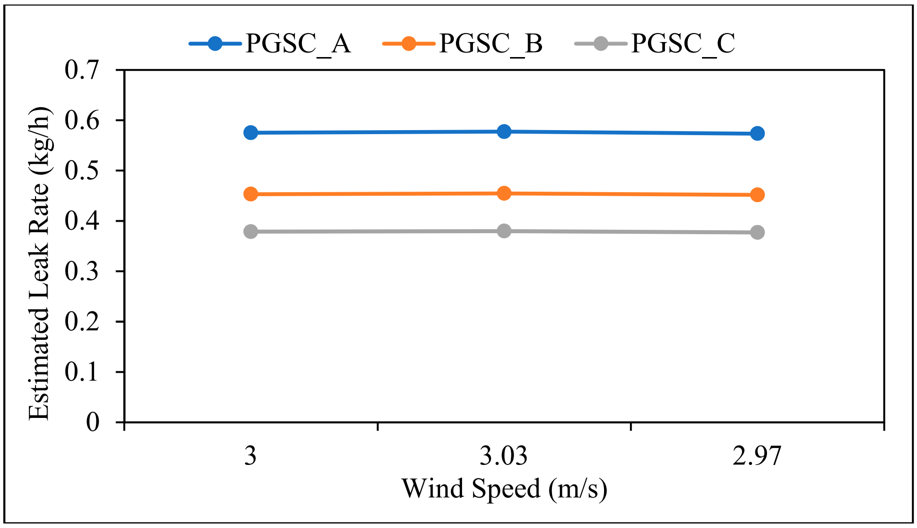

Figure S7: The estimated leak rates derived from varied wind speeds in PG stability classes A, B, and C.

S4.3: Uncertainty due to wind direction measurement

The wind direction determines the direction of flow of particles in the atmosphere; therefore, to calculate the surface emission in WindTrax, this was used as one of the inputs. To assess the impact the wind direction has on the estimated leak rate, an uncertainty analysis was carried out. The uncertainty associated with wind direction was calculated based on the sonic anemometer's accuracy of ±2°. The upper and lower bound of the wind direction was calculated as $WD_i \pm 2°$, where WD_i is the wind direction measurement. Input to WindTrax includes varied wind direction, varied stability classes, constant area of the source, constant wind speed, constant CH_4 mixing ratios, and constant downwind position of the RMLD. The surface emission rates were calculated in WindTrax and used to calculate the subsurface leak rates in the ESCAPE^{-1} model. A standard deviation was obtained for each PG stability class as $\sqrt{\frac{\sum_i^n (ELR_i - 0.4)^2}{(n-1)}}$, where ELR is the estimated leak rate and n is the number of data points in a stability class. Based on stability class B, the uncertainty associated with wind direction is (0.4±0.03kg/h).

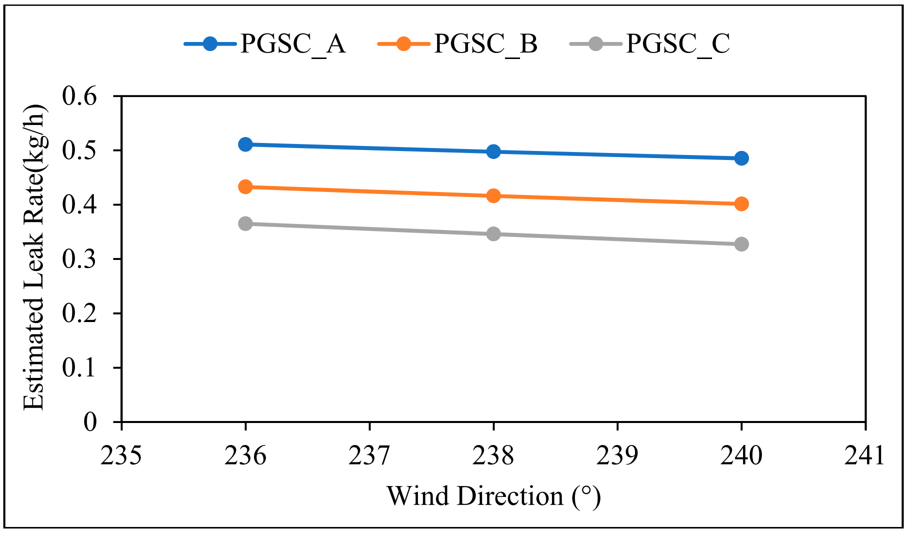

Figure S8: The estimated leak rates derived from varied wind directions in PG stability classes A, B, and C.

S4.4: Uncertainty due to classification of the Atmospheric Stability

Atmospheric stability is the tendency of the atmosphere to resist the vertical motion of air parcels. Measurements in this study were classified into three different stability classes (A, B, C, D) based on wind speed and daytime insolation (Pasquill (1961); Table S2). PG stability class A represents extremely unstable conditions, PG stability class B represents moderately unstable conditions and PG stability class C represents slightly unstable conditions. To illustrate the impact of this classification on the estimated leak rate, an uncertainty analysis was carried out.

In the uncertainty analysis, the PGSC was varied (A, B, C), while the wind speed, the wind direction, the area of the source, the CH_4 mixing ratios, and the downwind positioning of the RMLD were kept constant. The surface emission rates were calculated in WindTrax and used to calculate the subsurface leak rates in the ESCAPE^{-1} model. A standard deviation was obtained as $\sqrt{\frac{\sum_i^n (ELR_i - 0.4)^2}{(n-1)}}$, where ELR is the estimated leak rate and n is the number of data points. Based on this, the uncertainty associated with atmospheric stability is (0.4±0.12kg/h).

S4.5: Uncertainty due to the surface expression of a leak

A belowground leak forms an area source of emission on the surface. A study by Riddick et al., 2021 shows that gas migrates up to 4.5m from the leak center (the point directly above the leak). Therefore, to derive surface emissions in this modeling approach, we used a leak radius of 4.5 m.

To illustrate the uncertainty associated with the surface expression of a leak, the radius of the area source was varied from 0.5 to 5m, with 0.5m increments in WindTrax. The stability classes were varied, but the wind direction, the CH_4 mixing ratios, and the downwind position of the RMLD were kept constant. The surface emission rates were calculated in WindTrax and used to calculate the subsurface leak rates in the ESCAPE^{-1} model. A standard deviation was obtained for each PG stability class as $\sqrt{\frac{\sum_i^n (ELR_i - 0.4)^2}{(n-1)}}$, where ELR is the estimated leak rate, and n is the number of data points in a stability class. Based on stability class B, the uncertainty associated with the surface expression of a leak is (0.4±0.15kg/h).

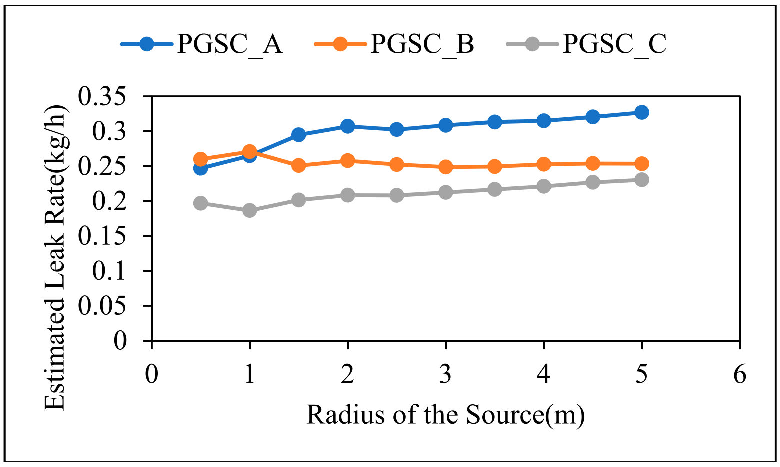

Figure S9: The estimated leak rates derived from varying the radius of the source from 0 to 5m in 0.5m increments. The estimated leak rates were calculated in PG stability classes A, B, and C.

S4.6: Propagation of Uncertainty

The uncertainty in the input data is propagated to the estimated below-ground leak rate. The estimated leak rate (ELR) in this approach is considered a function of changing line-averaged CH_4 mixing ratios (M), changing wind speed (WS), changing wind direction (WD), the surface expression of a leak (Ar), and classification of atmospheric stability to PG stability classes A, B, and C. This is represented as ELR = f (M, WS, WD, Ar, A, B, C).

Using data from the 0.4 kg h^{-1} leak, a linear model was used to correlate the estimated leak rate and the sources of uncertainty (Table S5). Each of the derivatives/estimates in each linear relationship were used to propagate the overall uncertainty (Equation S13). Based on the 0.4kg h^{-1} leak, the overall uncertainty in the modeling approach is (0.4±0.10kg/h).

$$\sigma_{ELR}^2 = \left(\frac{\delta(ELR)}{\delta(M)}\right)^2 \sigma_M^2 + \left(\frac{\delta(ELR)}{\delta(WS)}\right)^2 \sigma_{WS}^2 + \left(\frac{\delta(ELR)}{\delta(WD)}\right)^2 \sigma_{WD}^2 + \left(\frac{\delta(ELR)}{\delta(A)}\right)^2 \sigma_A^2 + \left(\frac{\delta(ELR)}{\delta(P)}\right)^2 \sigma_P^2 \quad (S13)$$

Table S5: Derivatives and standard deviation of each cause of uncertainty in the modeling approach. The product d^2*V^2 represents the derivative multiplied by the variance of each variable.

Variable	Derivative	Std (kg h^{-1})	Derivative² (d^2)	Variance (V^2)	d^2*V^2
M	0.007	0.20	0.000049	0.04	0.000002
WS	0.117	0.07	0.014	0.0049	0.00007
WD	-0.007	0.03	0.000049	0.0009	0.00000004
Ar	-0.0024	0.15	0.0000058	0.023	0.0000001
PG_A	-0.464	0.20	0.215	0.039	0.008
PG_B	-0.262	0.15	0.069	0.021	0.0015
PG_C	-0.12	0.16	0.014	0.026	0.0004
				Var (ELR)	0.01
				Std (ELR)	0.10

<u>Supplementary Material Section 5: Linear Relationships between the Variables and the Estimated Leak Rate</u>

S5.1: Methane Measurements and the Estimated Leak Rates

Figure S10 shows the linear relationship between CH_4 mixing ratios (M), and the estimated leak rate.

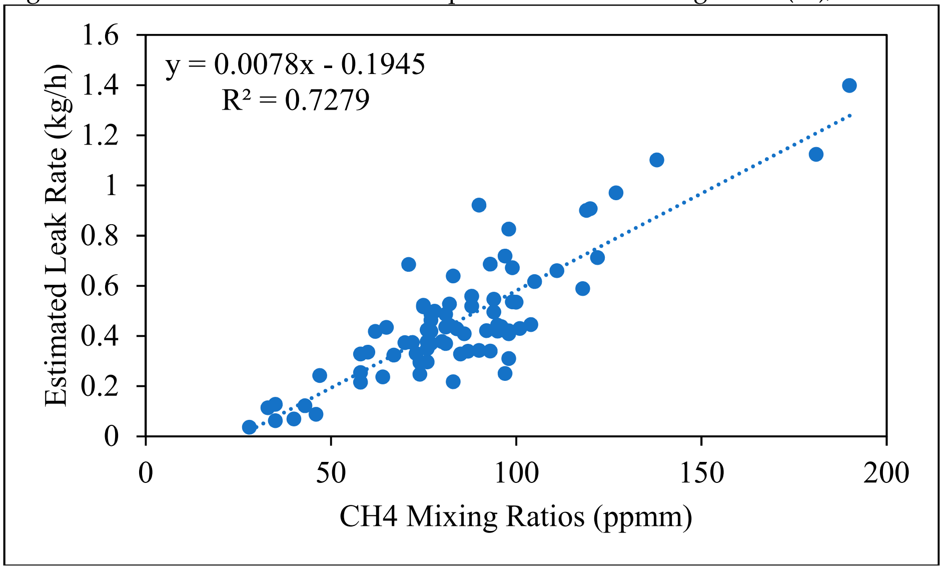

Figure S10: A linear relationship between the estimated leak rate and methane mixing ratios.

S5.2: Wind speed and the estimated leak rates

Figure S11 shows a linear relationship between wind speed and the estimated leak rate.

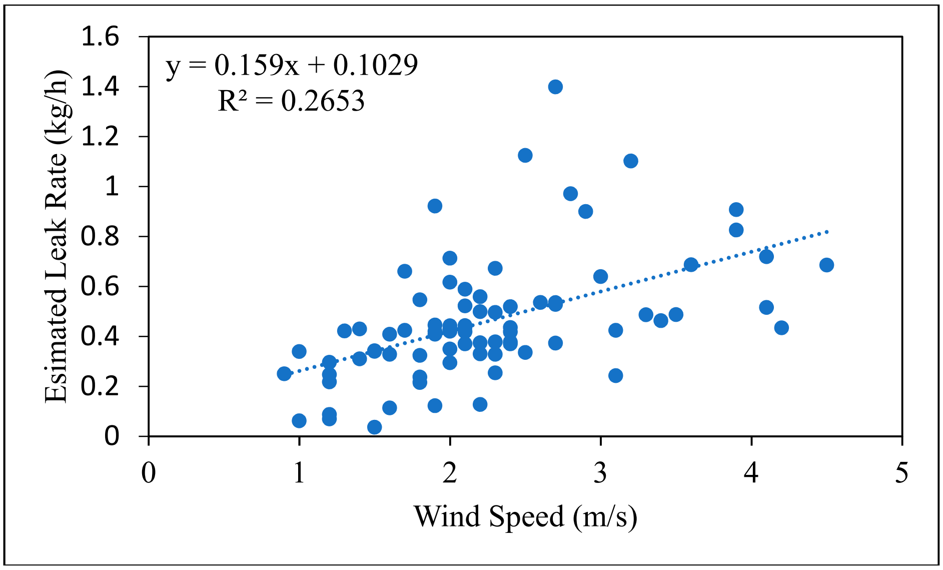

Figure S11: A linear relationship between the estimated leak rate and wind speed

S5.3: Wind direction and the estimated leak rates

Figure S12 below shows a linear relationship between the wind direction and the estimated leak rate.

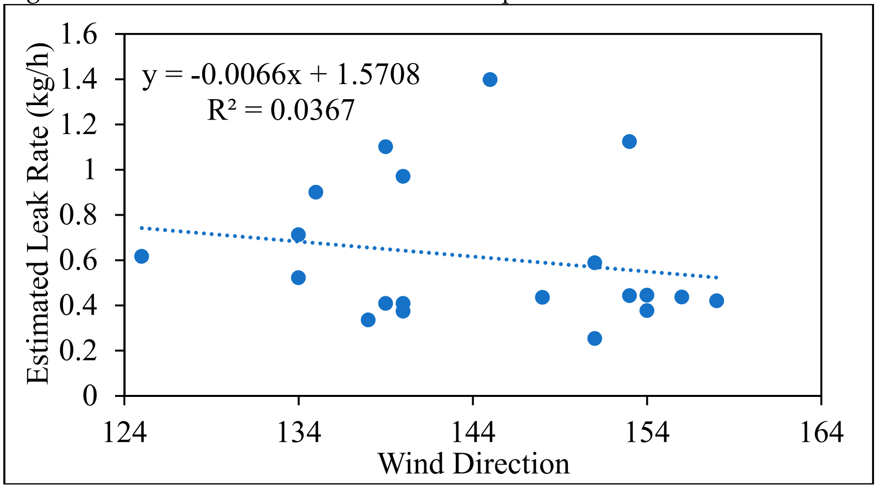

Figure S12: A linear relationship between the estimated leak rate and wind direction

S5.4: Area of the source and the estimated leak rates

When deriving the surface emission in WindTrax, the radius of the source was constantly considered to be 4.5m. Therefore, using this to establish a relationship with the estimated leak rate was not possible. To establish a linear relationship with the estimated leak rate, the results of section S4.5 were used. Figure S13 shows a linear relationship between the radius of the source and the estimated leak rate.

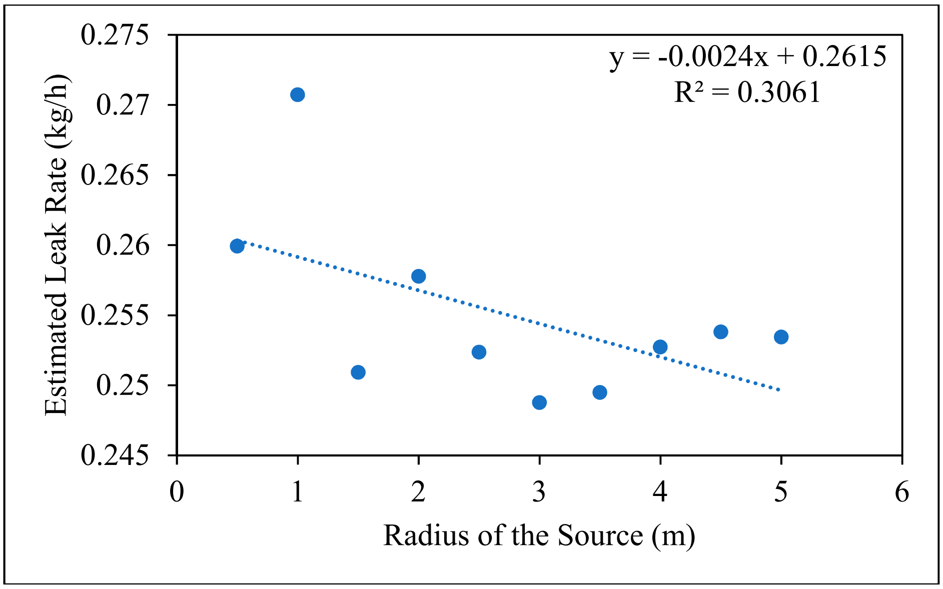

Figure S13: A linear relationship between the estimated leak rate and the radius of the source

Supplementary Material Section 6: Aboveground Plume

In this study, we used a line-averaging sensor to take aboveground CH_4 measurements. This means that to obtain reliable measurements, the sensor should be set up downwind and perpendicular to the wind direction. The essence of this setup is to ensure that the sensor is averaging across the plume centerline. A plume centerline is the center of mass of gas particles, i.e., a high concentration of particles (Figure S14). From the centerline, the concentrations of gas particles decrease (to approximately 10% of the concentrations at the centerline) as you move toward the plume edge.

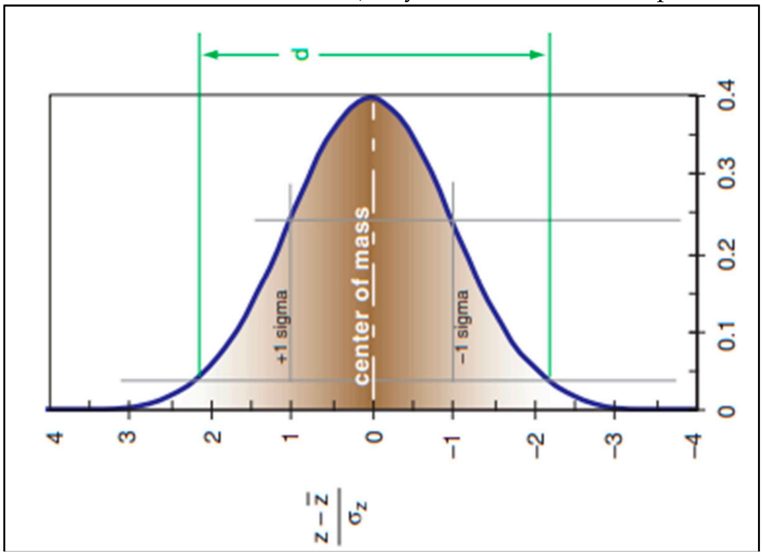

Figure S14: Dispersion of gas in the atmosphere from the plume 'centerline' (Centre of mass, Stull (2015)

The modeling approach in this study was biased when the wind direction changed rapidly. This is because the change in wind direction shifts the gas plume and makes the sensor measure either along the plume centerline or at the plume edge. If measuring along the plume centerline, then it is measuring high concentrations. If measuring at the plume edge, then it is measuring low concentrations of methane. Measuring from either the plume centerline or the plume edge biases the belowground estimated leak rate (by overestimating or underestimating, respectively).

Supplementary Material Section 7: Wind Speed and Wind Direction

Figure S15 shows wind speeds and their respective wind direction for the experiments conducted.

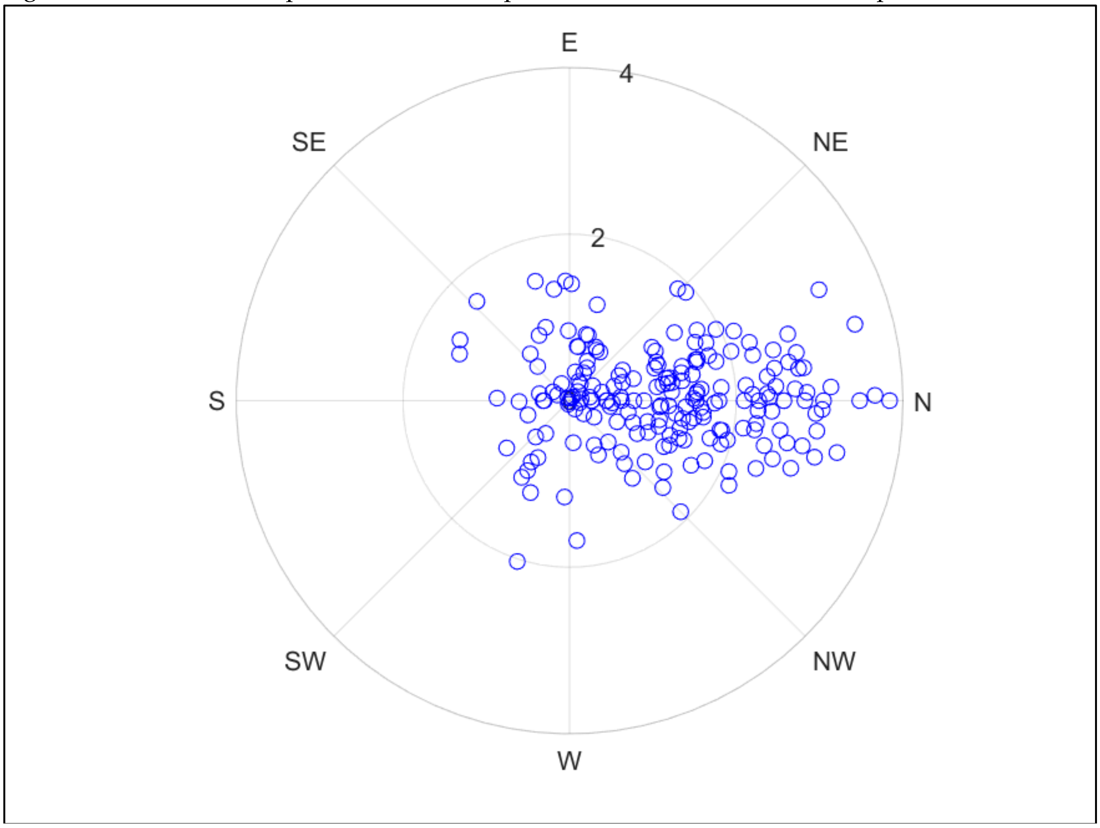

Figure S15: Wind speed rose for the METEC facility between September and December 2021. Wind speed ranged from 0.7 to less than 5 m s^{-1}.

Supplementary Material Section 8: Temperature and Relative Humidity

The results of a Pearson's correlation test show that temperature and relative humidity do not have a linear correlation with the estimated leak rate (Table S6).

Table S6: Results of Pearson's correlation between the estimated leak rate, temperature, and relative humidity.

	p-value	R^2	m
Temperature	<0.05	0.1043	-0.0120
Relative Humidity	<0.05	0.0148	0.0020
